# Supplementary material for: Genomic Insights into Cultivated Mexican Vanilla planifolia Reveal High Levels of Heterozygosity Stemming from Hybridization
Source: Plants (Basel). 2022 Aug 11;11(16):2090. doi: 10.3390/plants11162090 (PMC9412680; doi:10.3390/plants11162090)
Supplement: Supplementary file 1 [file plants-11-02090-s001.zip › Figure S1.pdf]

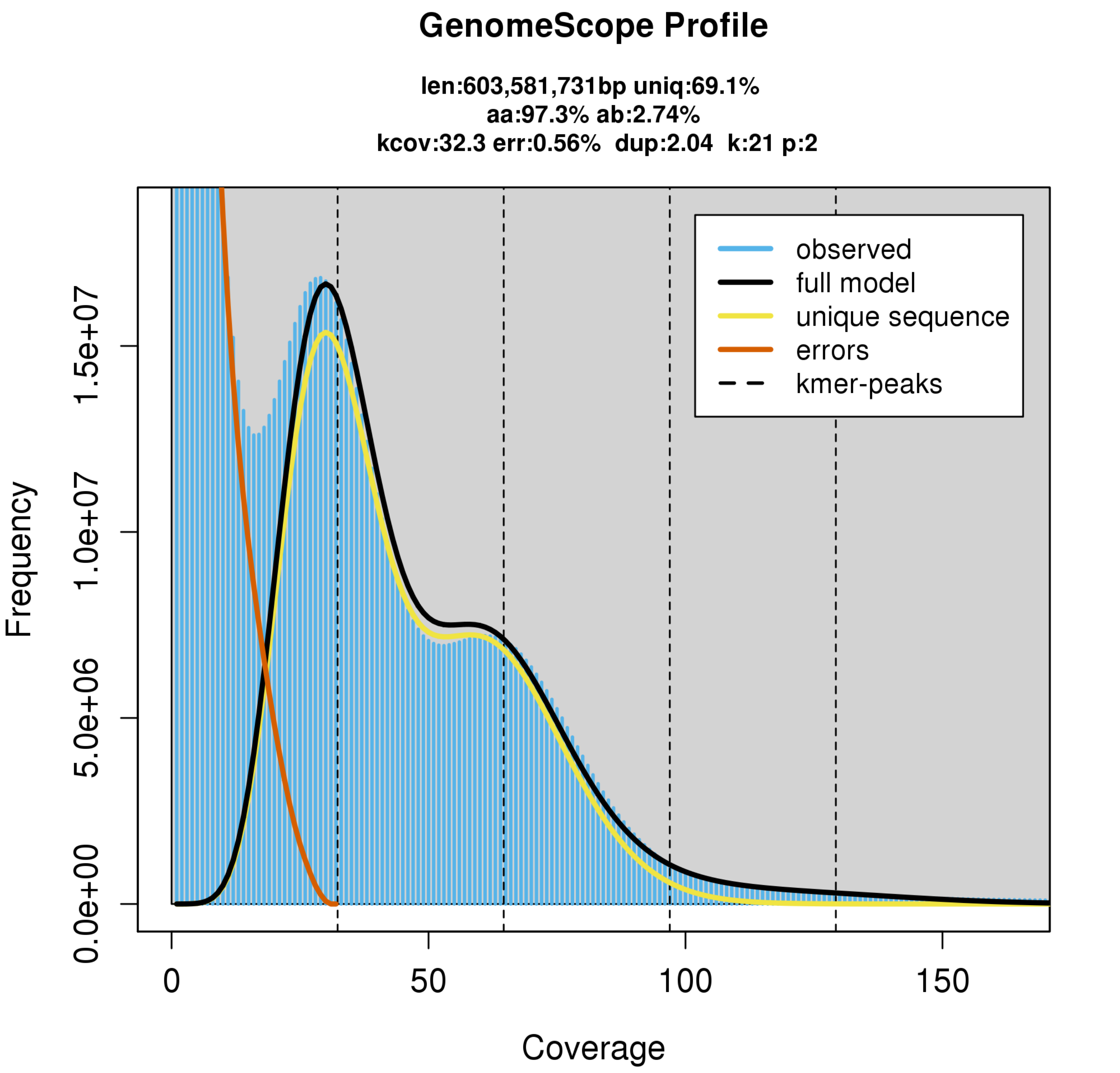
GenomeScope Profile
len:603,581,731bp uniq:69.1%
aa:97.3% ab:2.74%
kcov:32.3 err:0.56% dup:2.04 k:21 p:2
observed
full model
unique sequence
errors
kmer-peaks
Frequency
0.0e+00
5.0e+06
1.0e+07
1.5e+07
Coverage
0
50
100
150

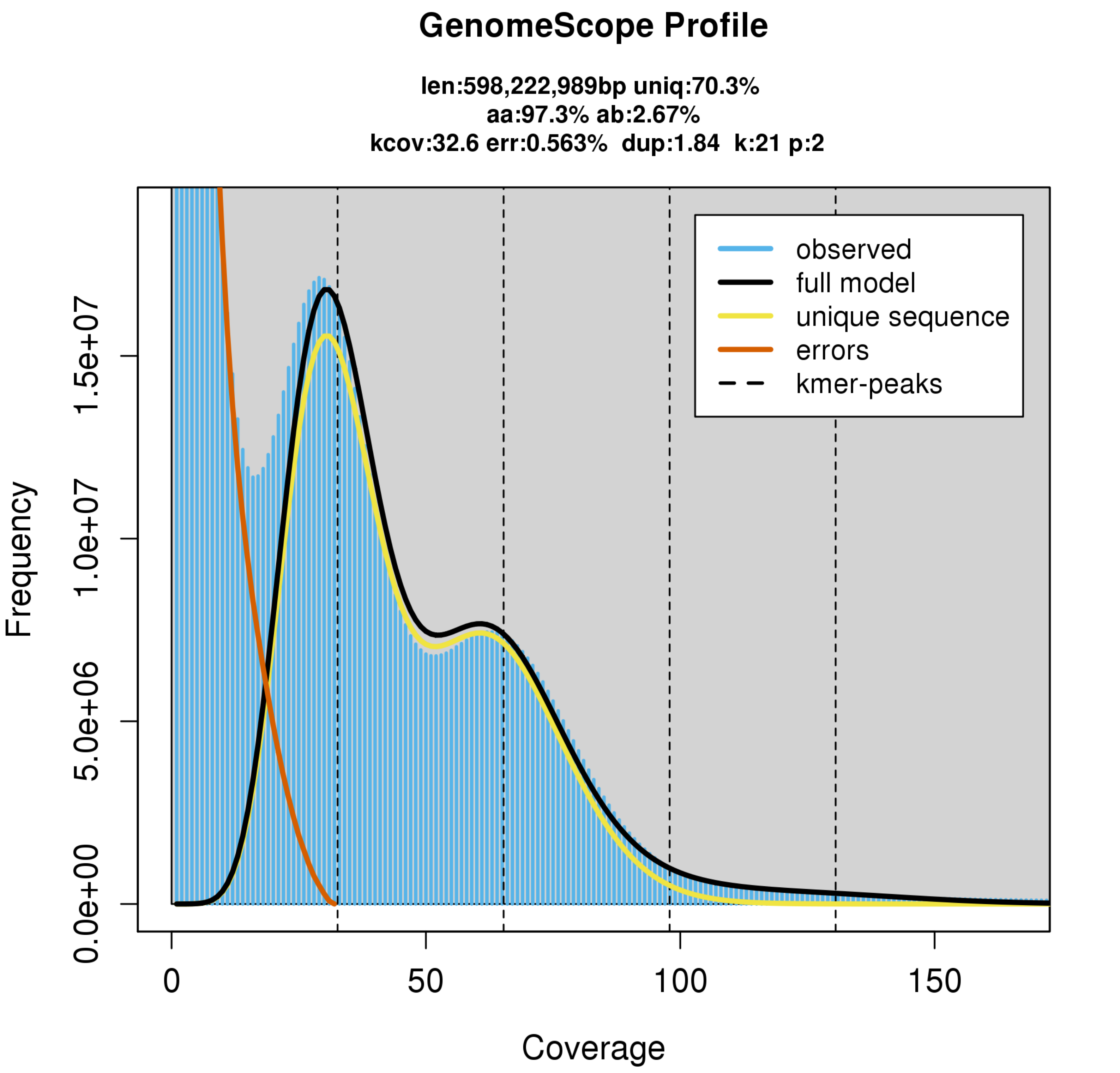
GenomeScope Profile
len:598,222,989bp uniq:70.3%
aa:97.3% ab:2.67%
kcov:32.6 err:0.563% dup:1.84 k:21 p:2
observed
full model
unique sequence
errors
kmer-peaks
Frequency
0.0e+00
5.0e+06
1.0e+07
1.5e+07
Coverage
0
50
100
150

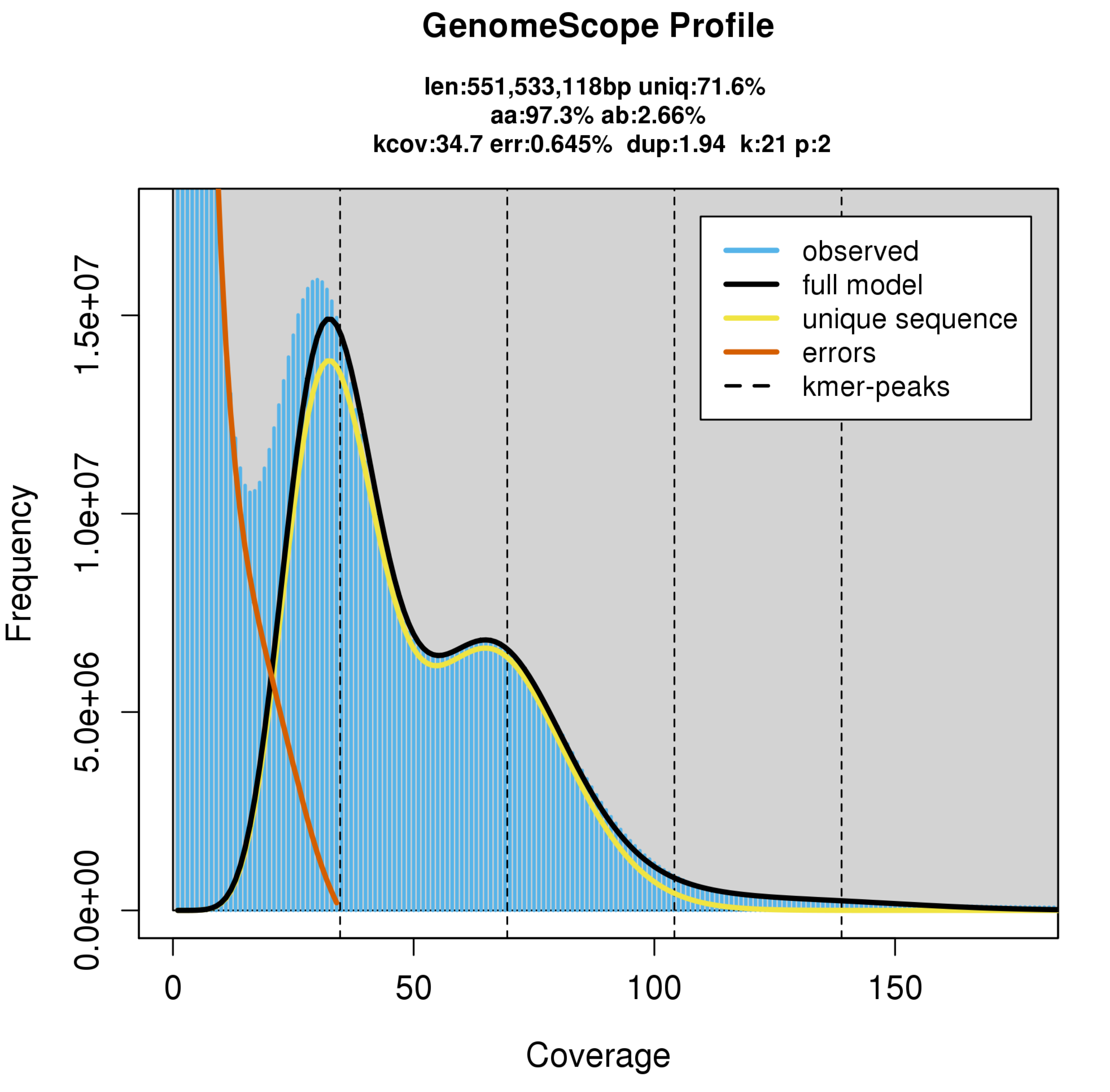
GenomeScope Profile
len:551,533,118bp uniq:71.6%
aa:97.3% ab:2.66%
kcov:34.7 err:0.645% dup:1.94 k:21 p:2
observed
full model
unique sequence
errors
kmer-peaks
Frequency
0.0e+00
5.0e+06
1.0e+07
1.5e+07
Coverage
0
50
100
150

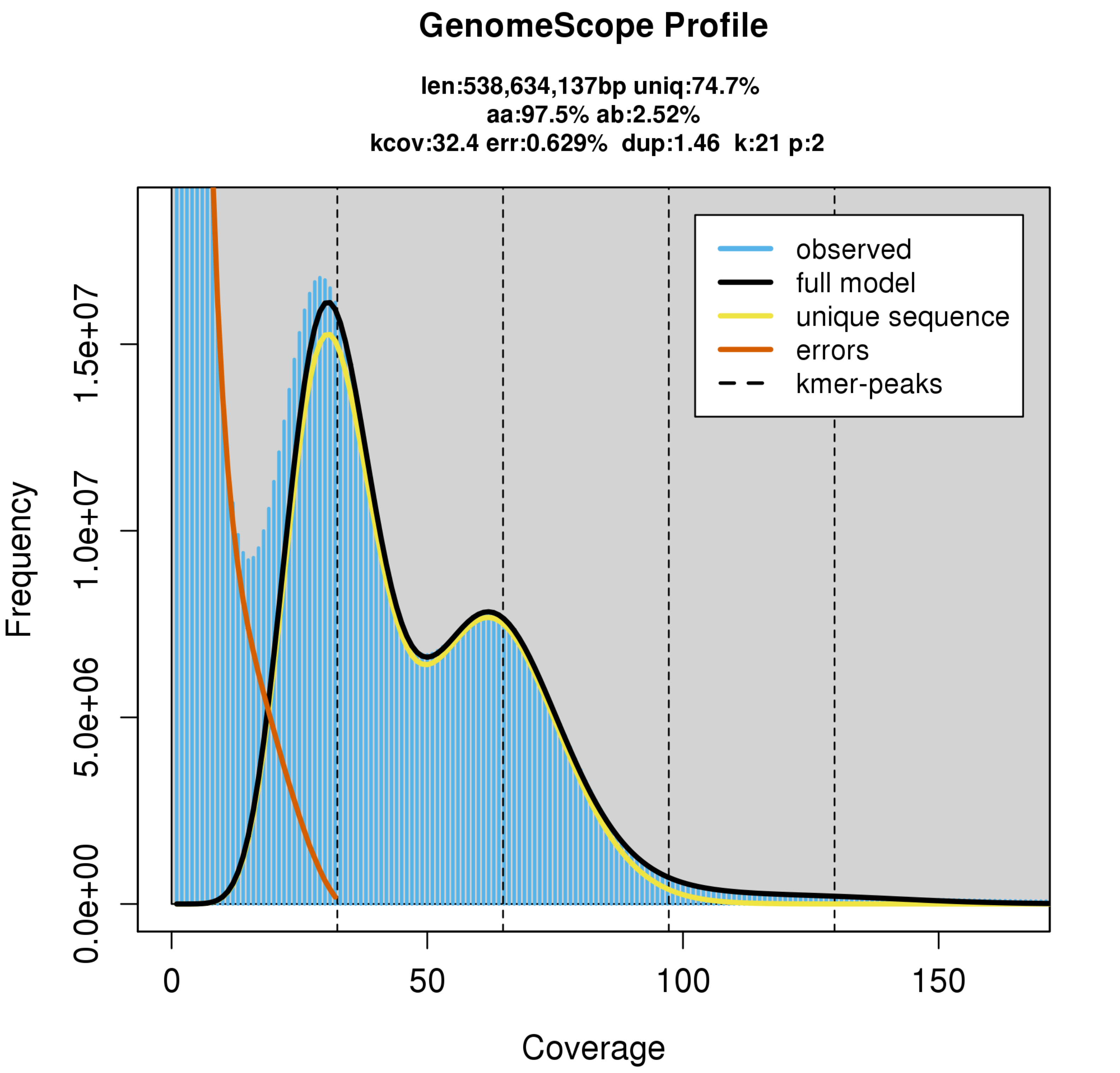
GenomeScope Profile
len:538,634,137bp uniq:74.7%
aa:97.5% ab:2.52%
kcov:32.4 err:0.629% dup:1.46 k:21 p:2
observed
full model
unique sequence
errors
kmer-peaks
Frequency
0.0e+00
5.0e+06
1.0e+07
1.5e+07
Coverage
0
50
100
150

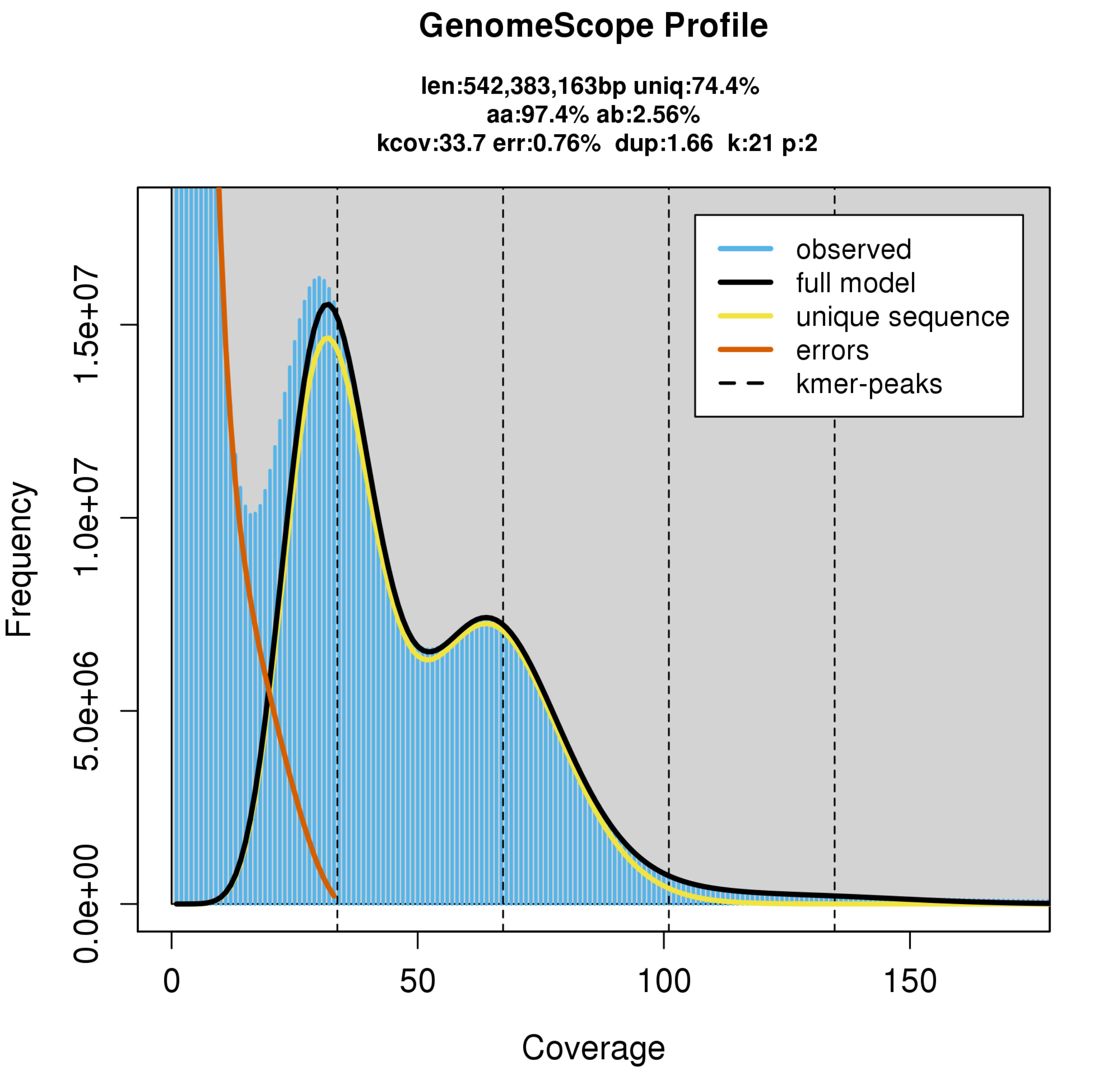

GenomeScope Profile
len:542,383,163bp uniq:74.4%
aa:97.4% ab:2.56%
kcov:33.7 err:0.76% dup:1.66 k:21 p:2
observed
full model
unique sequence
errors
kmer-peaks
Frequency
0.0e+00
5.0e+06
1.0e+07
1.5e+07
Coverage
0
50
100
150

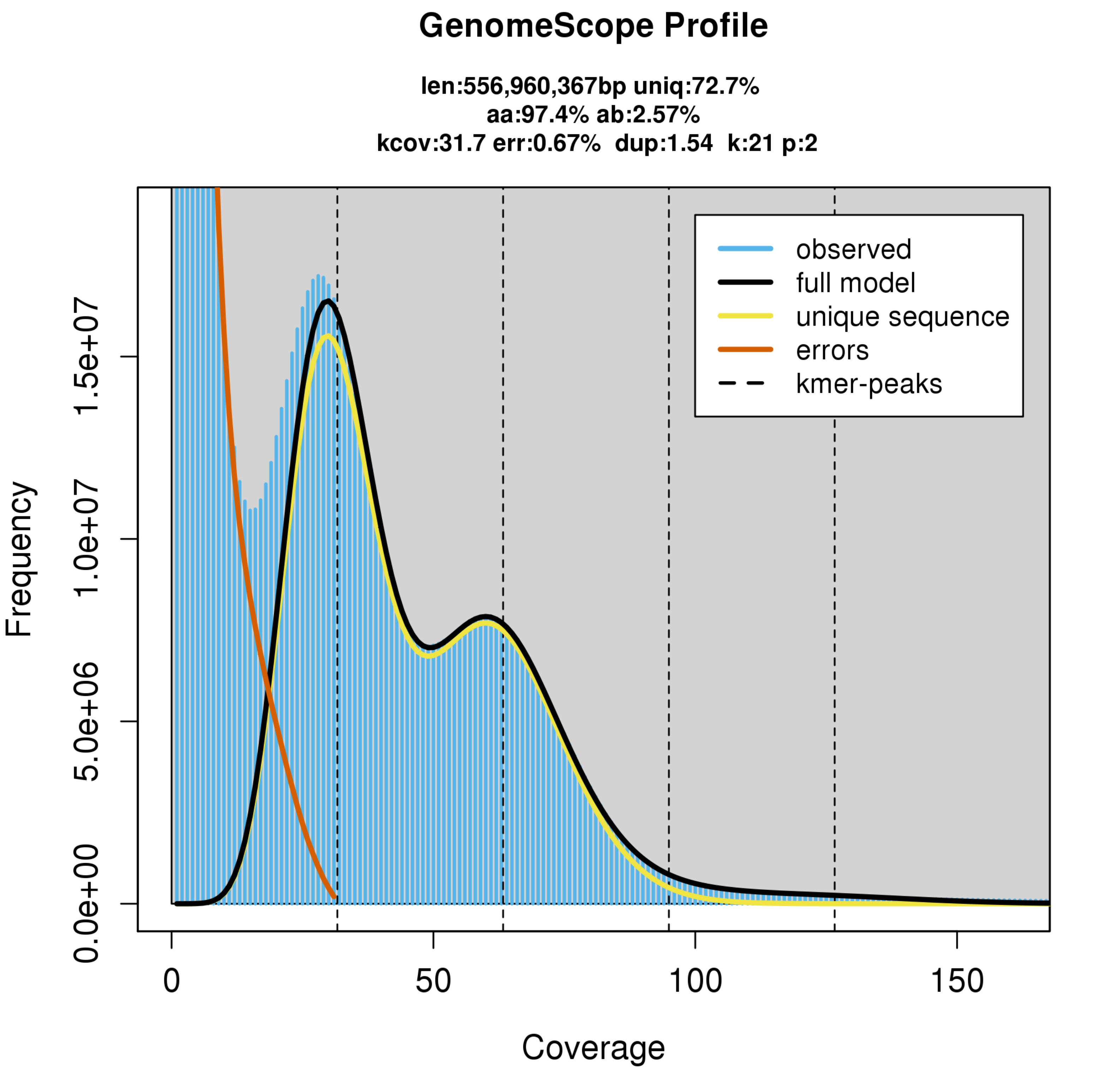
GenomeScope Profile
len:556,960,367bp uniq:72.7%
aa:97.4% ab:2.57%
kcov:31.7 err:0.67% dup:1.54 k:21 p:2
observed
full model
unique sequence
errors
kmer-peaks
Frequency
0.0e+00
5.0e+06
1.0e+07
1.5e+07
0
50
100
150
Coverage

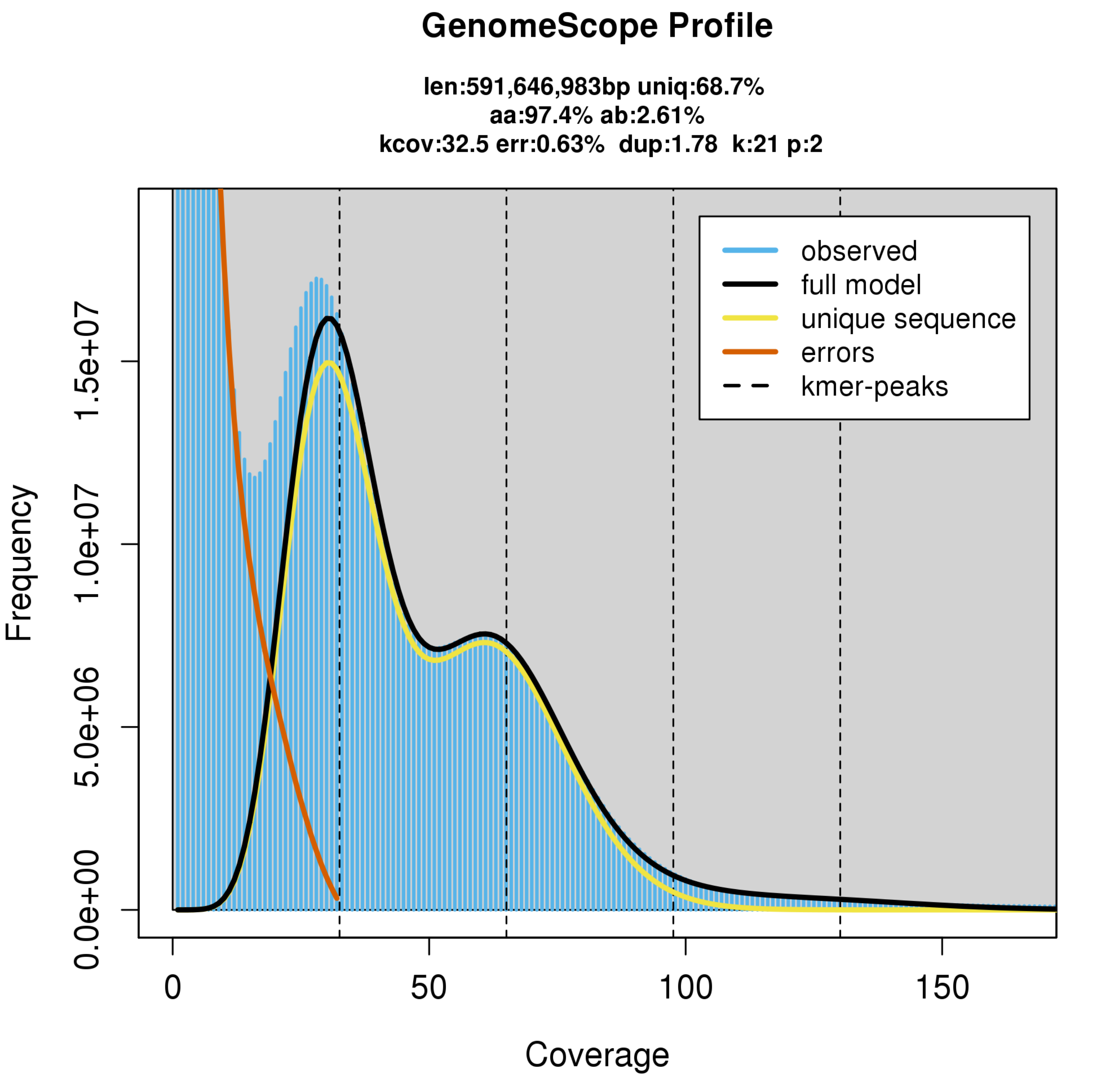
GenomeScope Profile
len:591,646,983bp uniq:68.7%
aa:97.4% ab:2.61%
kcov:32.5 err:0.63% dup:1.78 k:21 p:2
observed
full model
unique sequence
errors
kmer-peaks
Frequency
0.0e+00
5.0e+06
1.0e+07
1.5e+07
Coverage
0
50
100
150

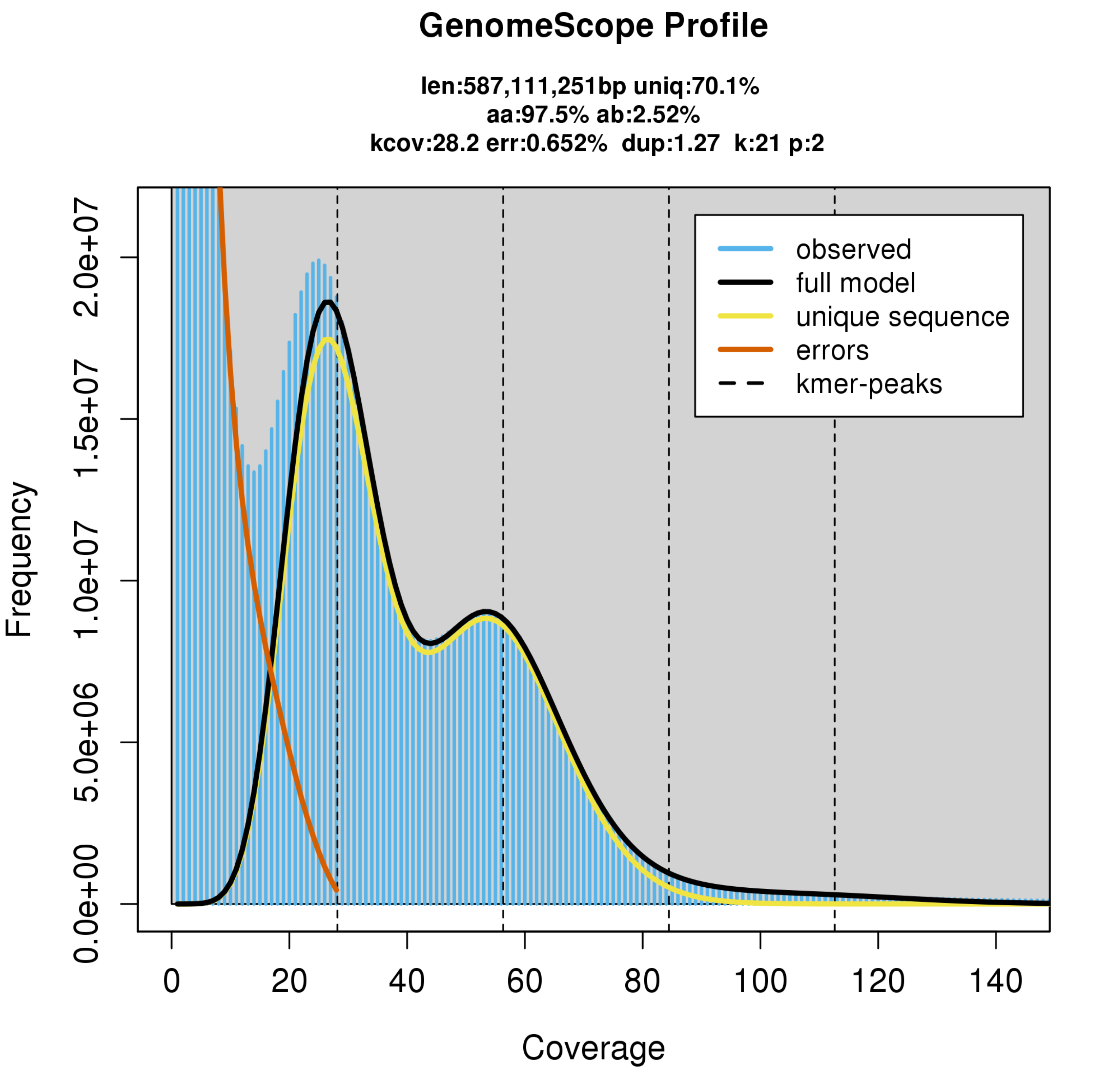

GenomeScope Profile
len:587,111,251bp uniq:70.1%
aa:97.5% ab:2.52%
kcov:28.2 err:0.652% dup:1.27 k:21 p:2
Frequency
0.0e+00
5.0e+06
1.0e+07
1.5e+07
2.0e+07
Coverage
0
20
40
60
80
100
120
140
observed
full model
unique sequence
errors
kmer-peaks

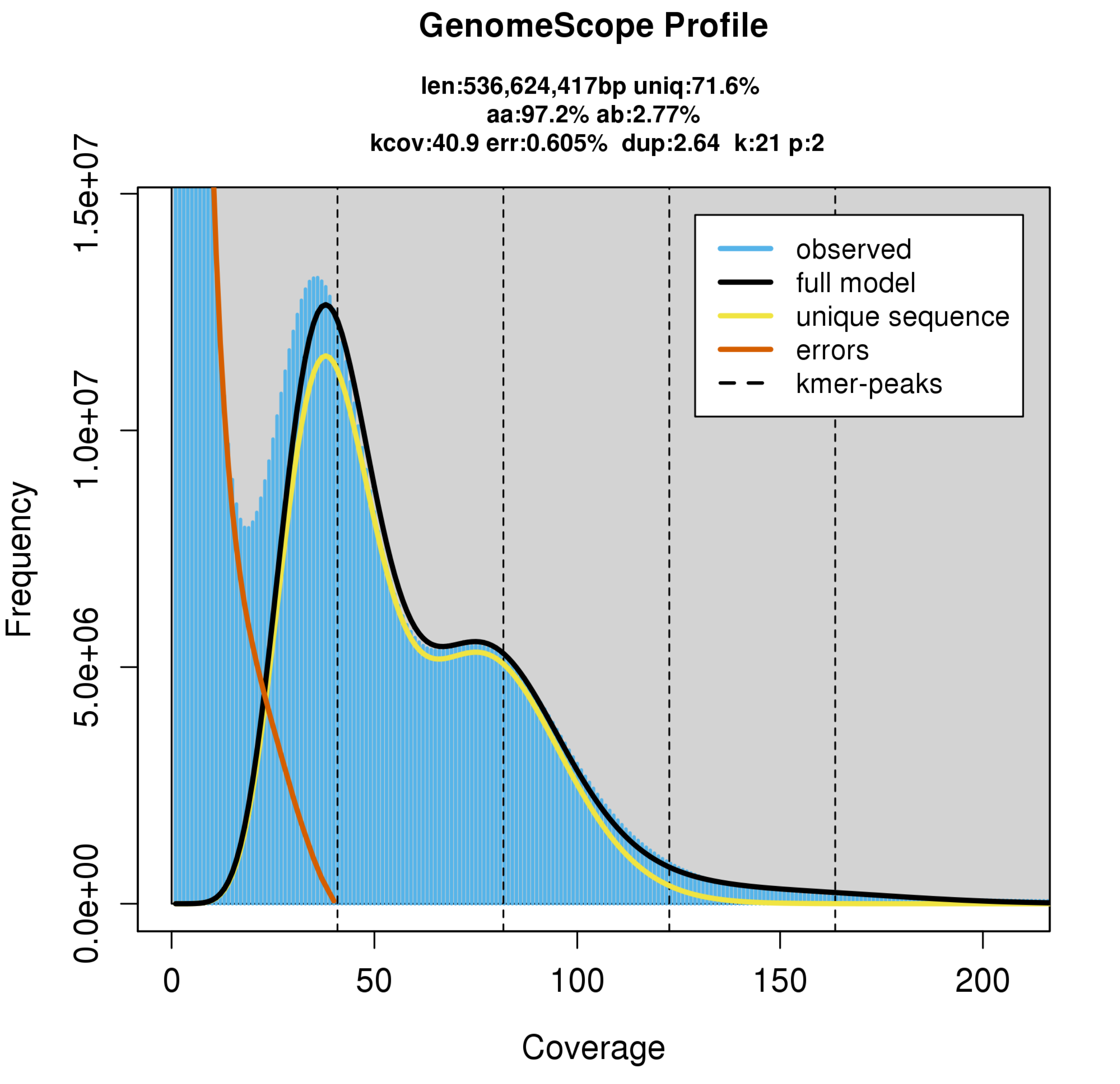
GenomeScope Profile
len:536,624,417bp uniq:71.6%
aa:97.2% ab:2.77%
kcov:40.9 err:0.605% dup:2.64 k:21 p:2
observed
full model
unique sequence
errors
kmer-peaks
Frequency
Coverage
0.0e+00
5.0e+06
1.0e+07
1.5e+07
0
50
100
150
200

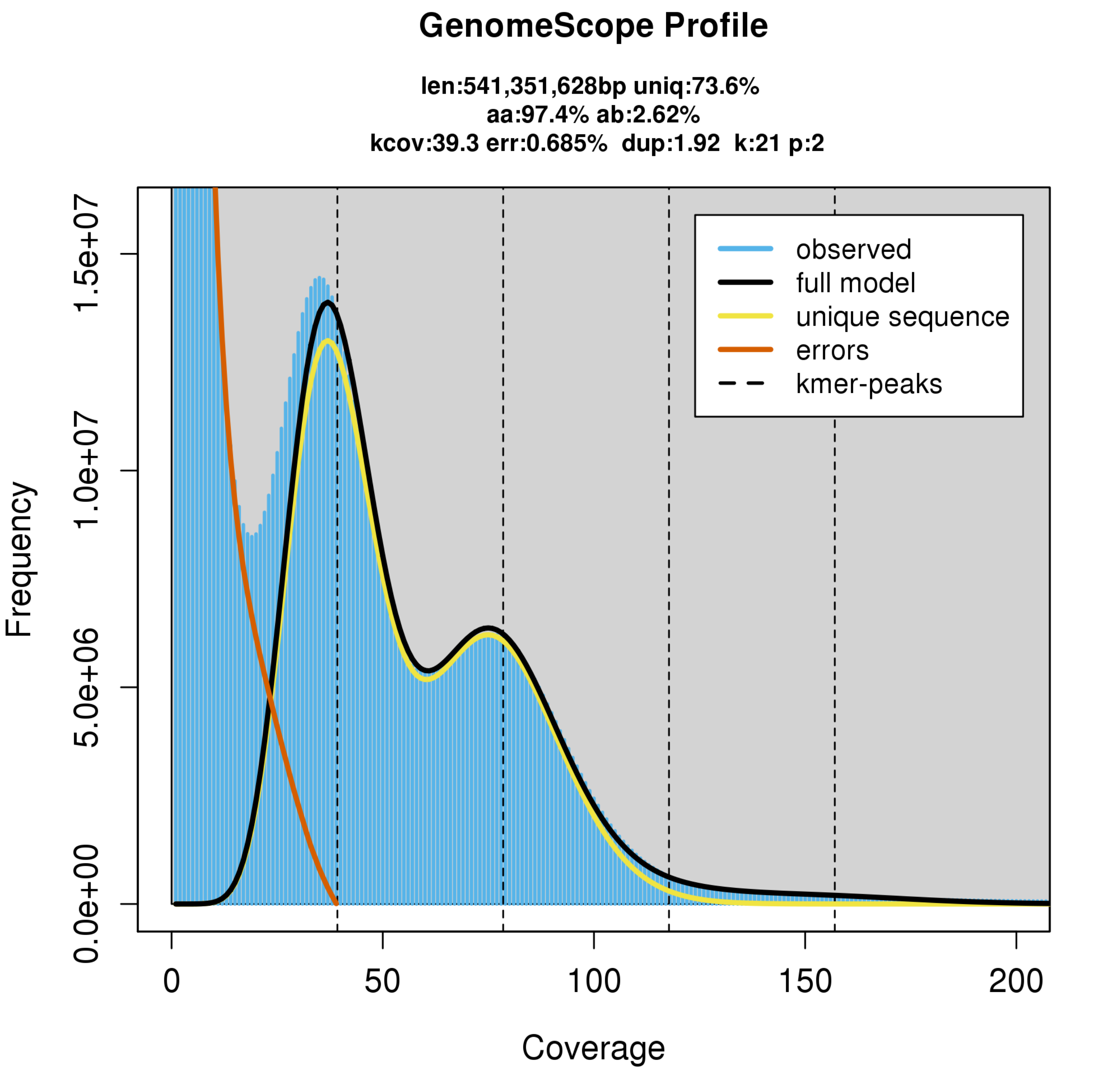
GenomeScope Profile
len:541,351,628bp uniq:73.6%
aa:97.4% ab:2.62%
kcov:39.3 err:0.685% dup:1.92 k:21 p:2
observed
full model
unique sequence
errors
kmer-peaks
Frequency
Coverage
0.0e+00
5.0e+06
1.0e+07
1.5e+07
0
50
100
150
200

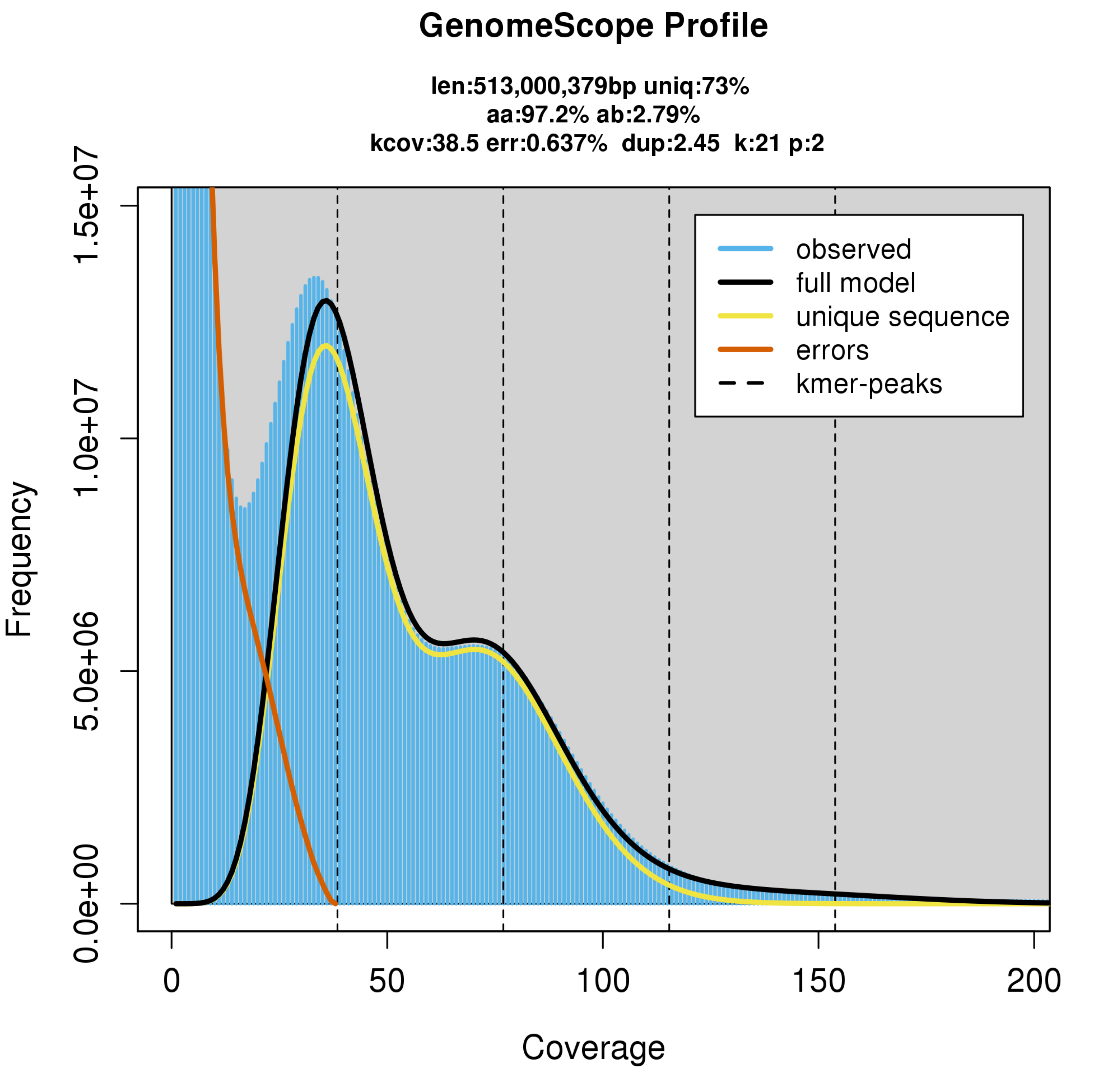
GenomeScope Profile
len:513,000,379bp uniq:73%
aa:97.2% ab:2.79%
kcov:38.5 err:0.637% dup:2.45 k:21 p:2
observed
full model
unique sequence
errors
kmer-peaks
Frequency
0.0e+00
5.0e+06
1.0e+07
1.5e+07
Coverage
0
50
100
150
200

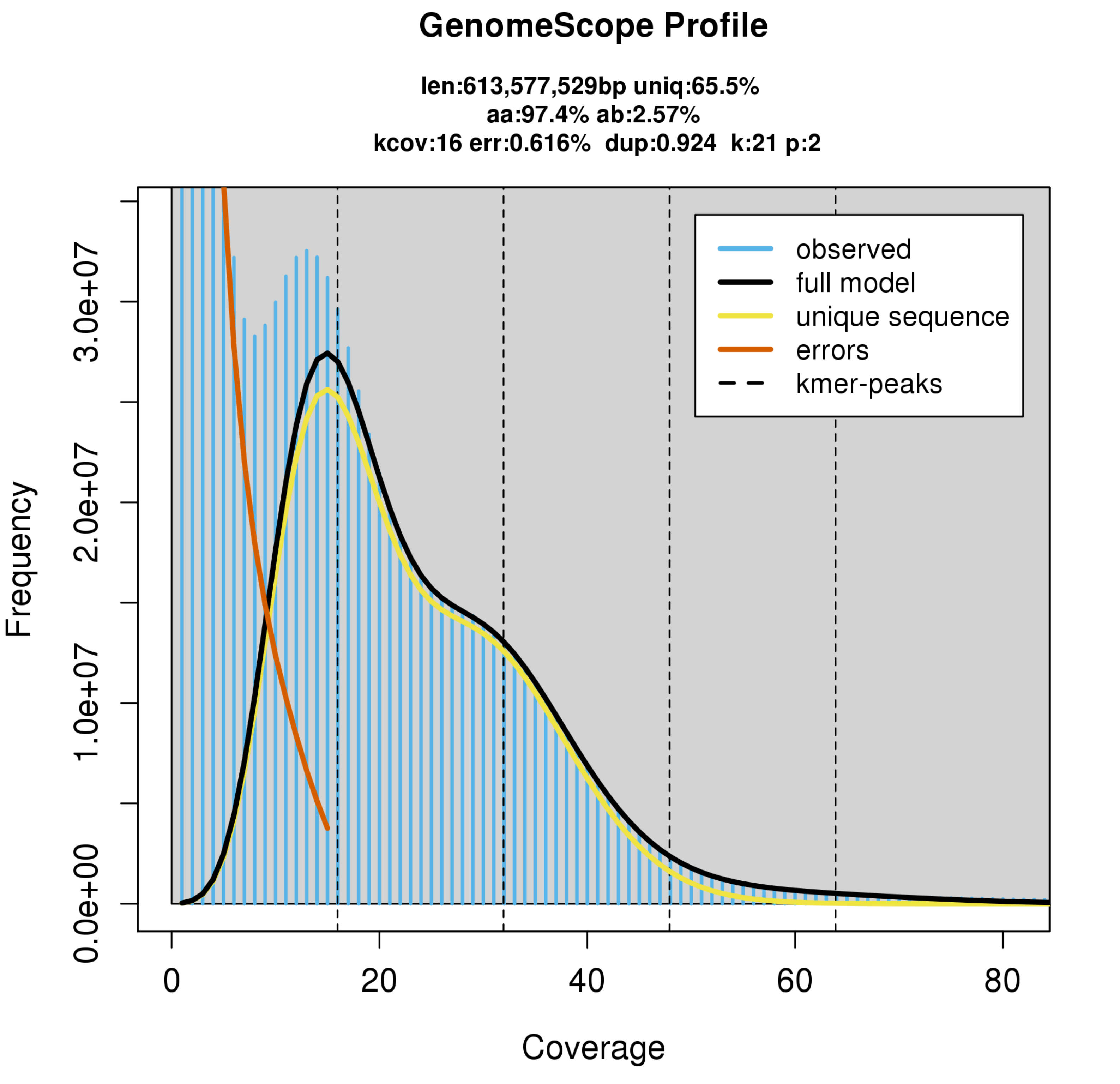

GenomeScope Profile
len:613,577,529bp uniq:65.5%
aa:97.4% ab:2.57%
kcov:16 err:0.616% dup:0.924 k:21 p:2
observed
full model
unique sequence
errors
kmer-peaks
Frequency
0.0e+00
1.0e+07
2.0e+07
3.0e+07
Coverage
0
20
40
60
80

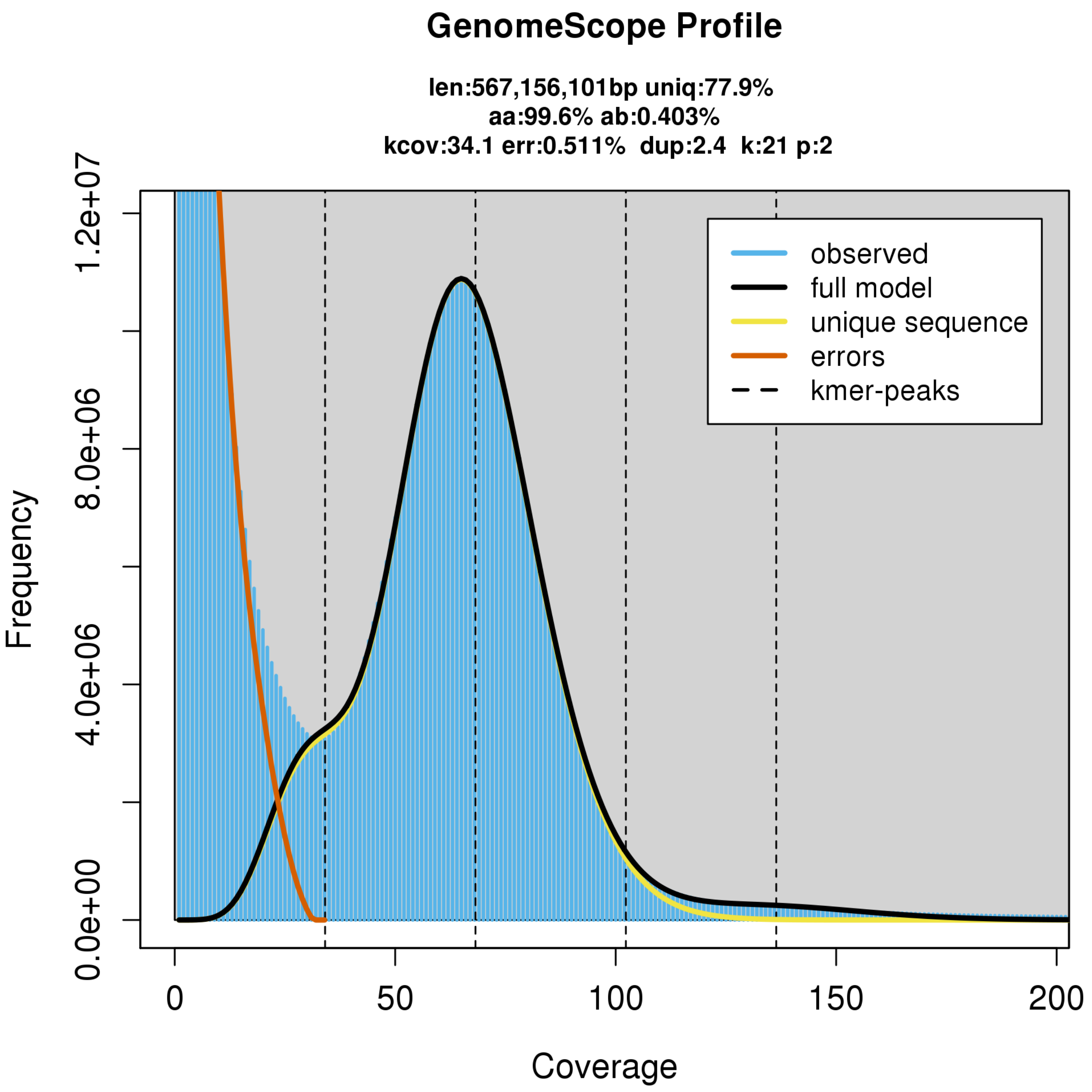
GenomeScope Profile
len:567,156,101bp uniq:77.9%
aa:99.6% ab:0.403%
kcov:34.1 err:0.511% dup:2.4 k:21 p:2
observed
full model
unique sequence
errors
kmer-peaks
Frequency
0.0e+00
4.0e+06
8.0e+06
1.2e+07
0
50
100
150
200
Coverage

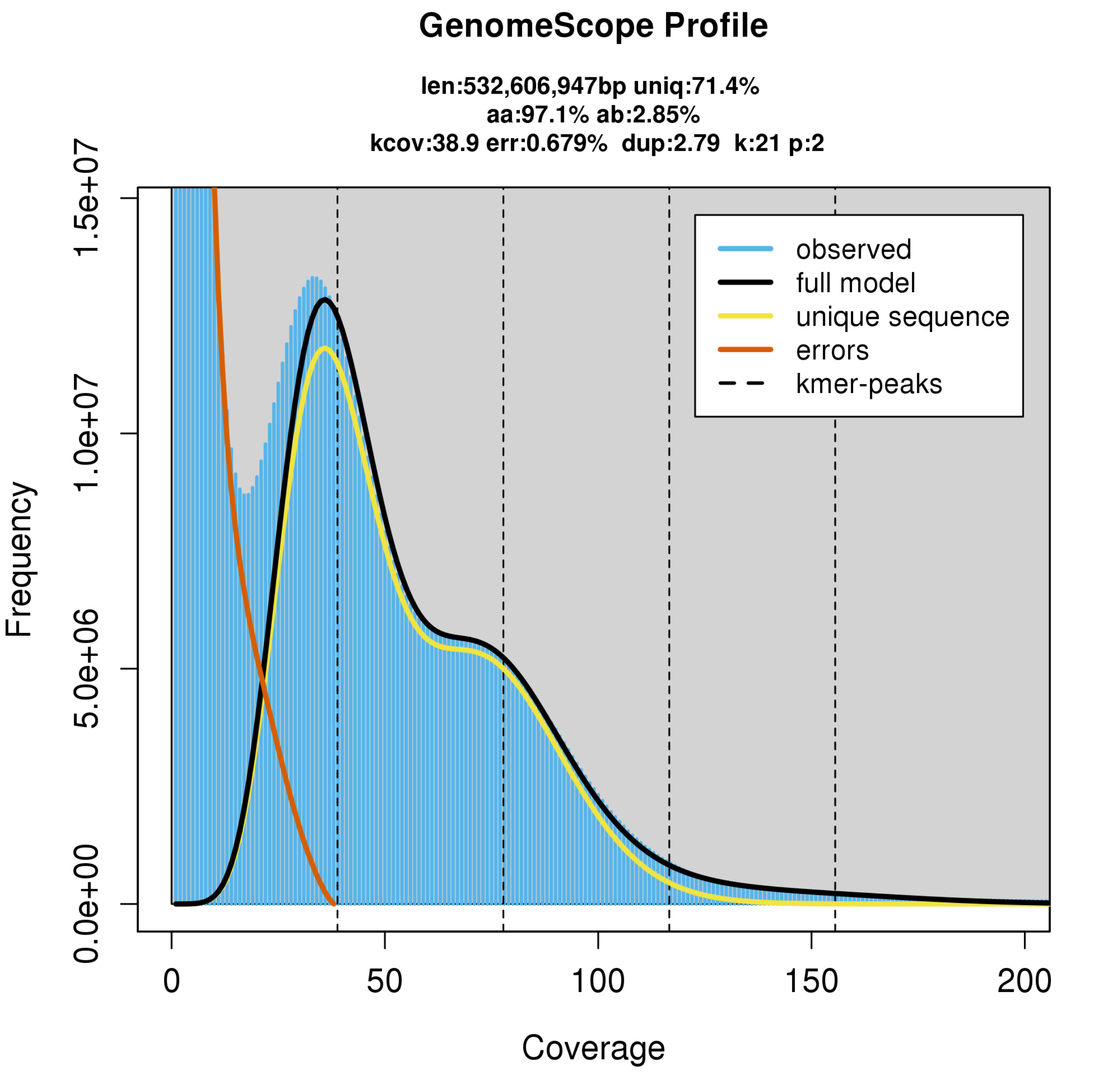

GenomeScope Profile
len:532,606,947bp uniq:71.4%
aa:97.1% ab:2.85%
kcov:38.9 err:0.679% dup:2.79 k:21 p:2
Frequency
0.0e+00
5.0e+06
1.0e+07
1.5e+07
Coverage
0
50
100
150
200
observed
full model
unique sequence
errors
kmer-peaks

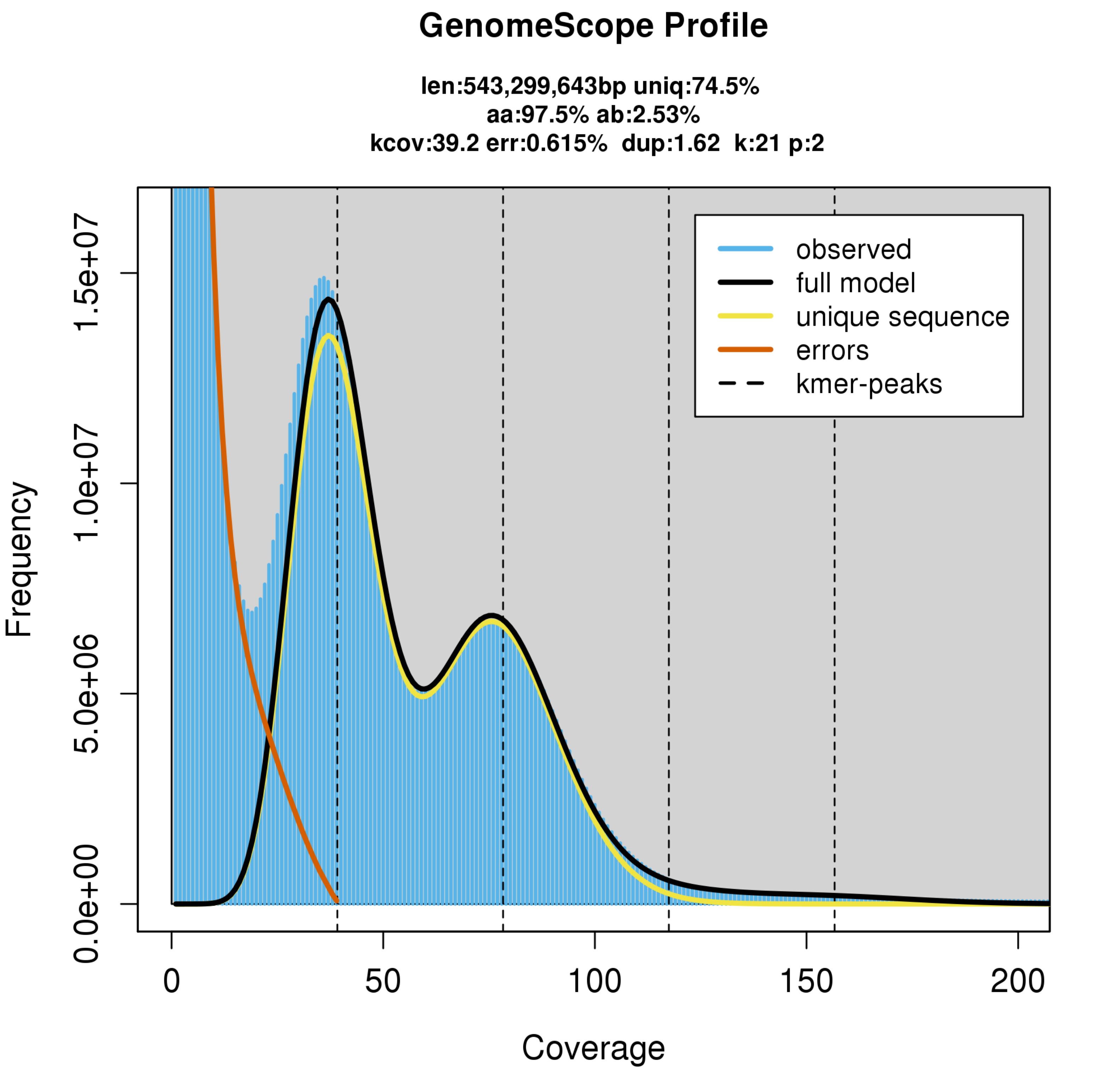

GenomeScope Profile
len:543,299,643bp uniq:74.5%
aa:97.5% ab:2.53%
kcov:39.2 err:0.615% dup:1.62 k:21 p:2
observed
full model
unique sequence
errors
kmer-peaks
Frequency
0.0e+00
5.0e+06
1.0e+07
1.5e+07
0
50
100
150
200
Coverage
