# Supplementary material for: Genomic Insights into Cultivated Mexican Vanilla planifolia Reveal High Levels of Heterozygosity Stemming from Hybridization
Source: Plants (Basel). 2022 Aug 11;11(16):2090. doi: 10.3390/plants11162090 (PMC9412680; doi:10.3390/plants11162090)
Supplement: Supplementary file 1 [file plants-11-02090-s001.zip › Figure S2.pdf]

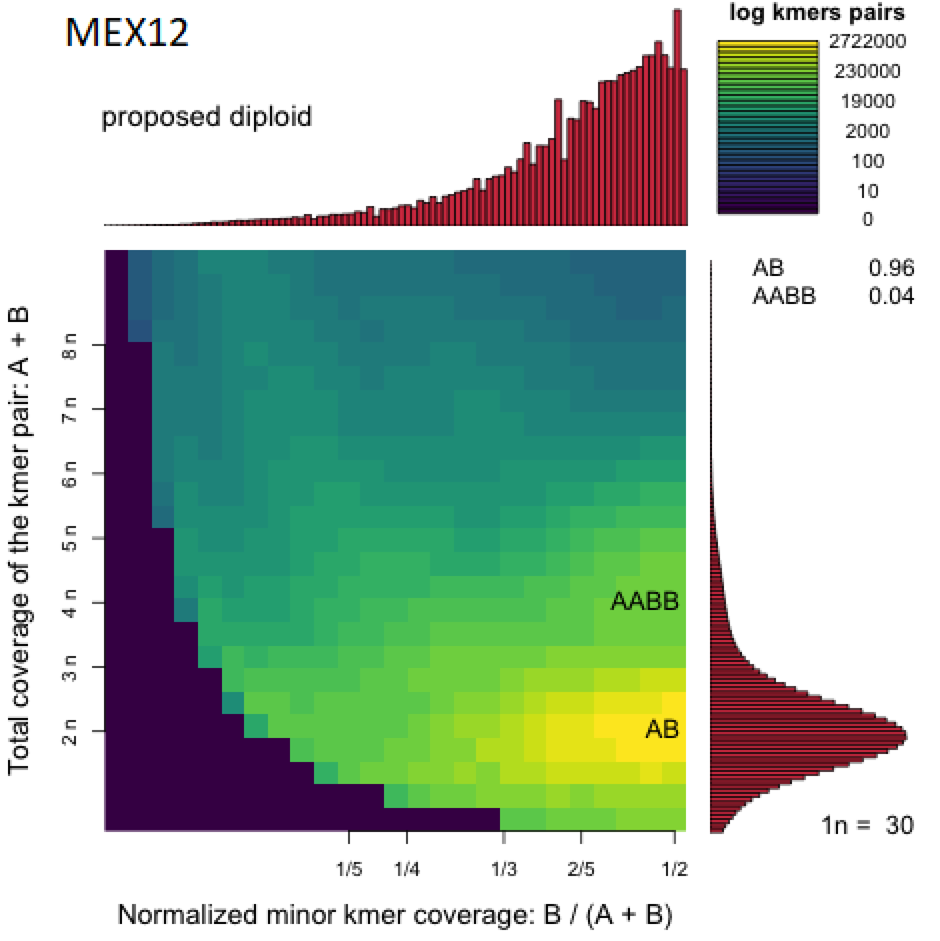
MEX12
proposed diploid
log kmers pairs
2722000
230000
19000
2000
100
10
0
AB 0.96
AABB 0.04
AABB
AB
1n = 30
Total coverage of the kmer pair: A + B
8n
7n
6n
5n
4n
3n
2n
1/5
1/4
1/3
2/5
1/2
Normalized minor kmer coverage: B / (A + B)

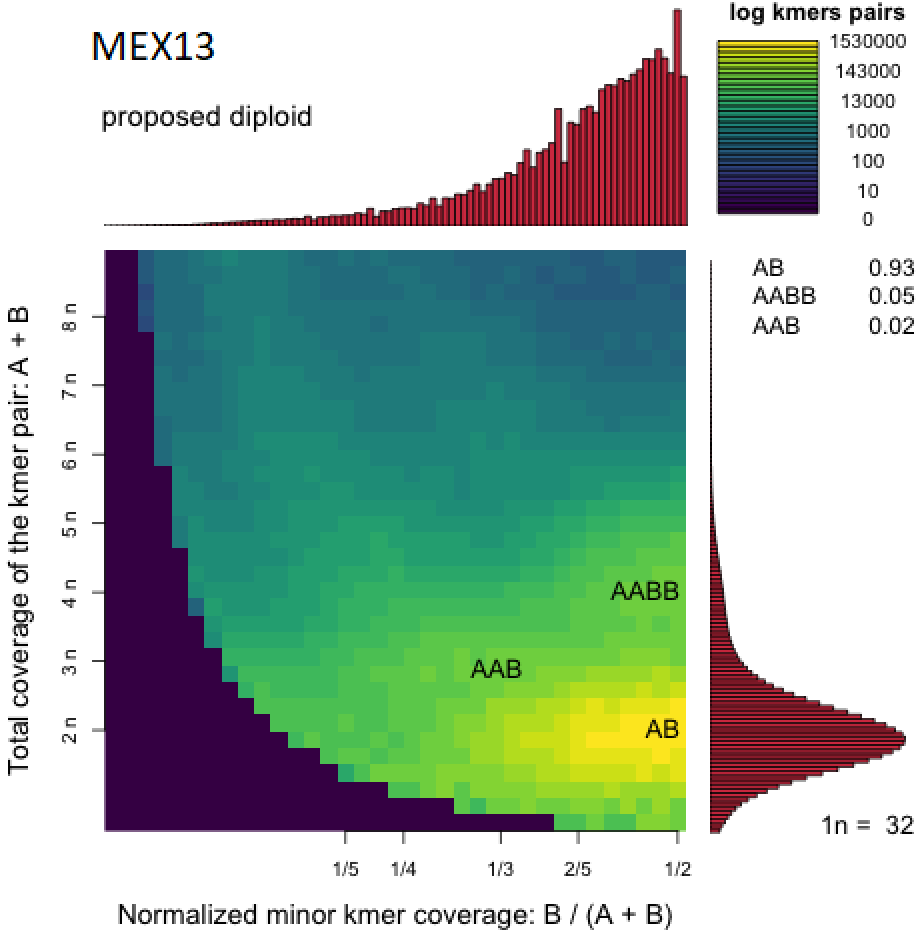
MEX13
proposed diploid
log kmers pairs
1530000
143000
13000
1000
100
10
0
AB 0.93
AABB 0.05
AAB 0.02
AABB
AAB
AB
1n = 32
Total coverage of the kmer pair: A + B
2n
3n
4n
5n
6n
7n
8n
1/5
1/4
1/3
2/5
1/2
Normalized minor kmer coverage: B / (A + B)

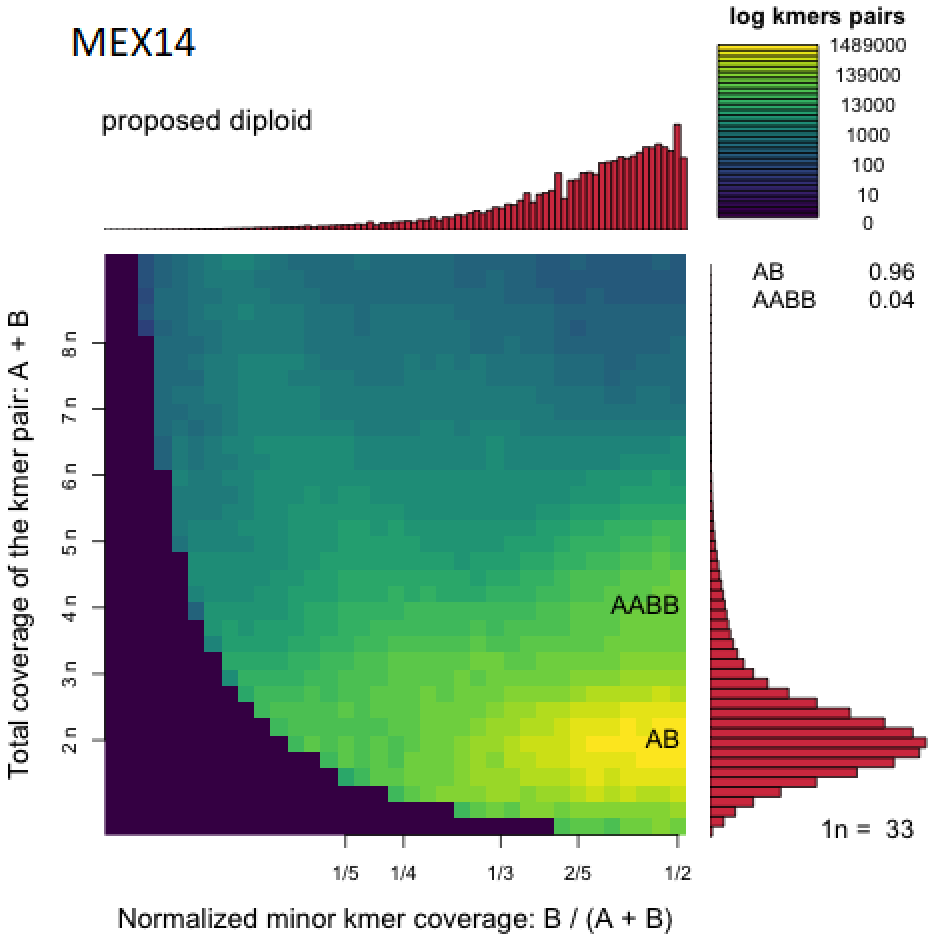
MEX14
proposed diploid
log kmers pairs
1489000
139000
13000
1000
100
10
0
AB 0.96
AABB 0.04
AABB
AB
1n = 33
Total coverage of the kmer pair: A + B
2n
3n
4n
5n
6n
7n
8n
1/5
1/4
1/3
2/5
1/2
Normalized minor kmer coverage: B / (A + B)

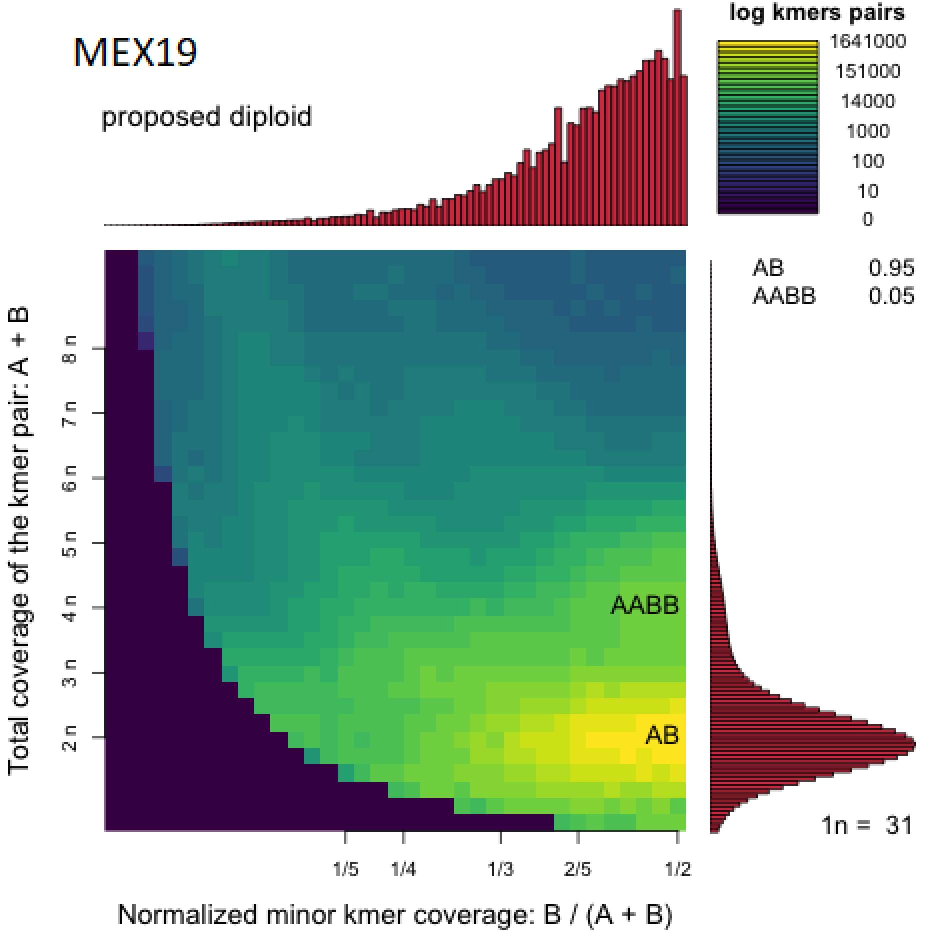
MEX19
proposed diploid
log kmers pairs
1641000
151000
14000
1000
100
10
0
AB 0.95
AABB 0.05
AABB
AB
1n = 31
Total coverage of the kmer pair: A + B
2n
3n
4n
5n
6n
7n
8n
1/5
1/4
1/3
2/5
1/2
Normalized minor kmer coverage: B / (A + B)

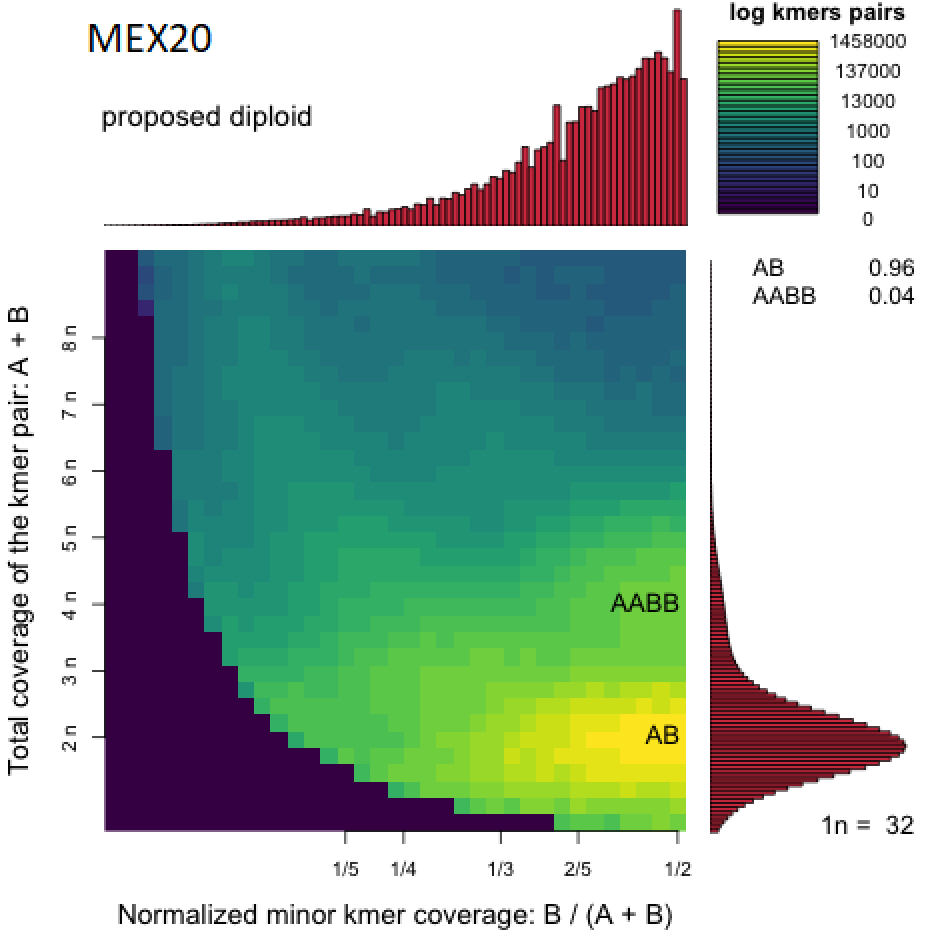
MEX20
proposed diploid
log kmers pairs
1458000
137000
13000
1000
100
10
0
AB 0.96
AABB 0.04
AABB
AB
1n = 32
Total coverage of the kmer pair: A + B
2n
3n
4n
5n
6n
7n
8n
1/5
1/4
1/3
2/5
1/2
Normalized minor kmer coverage: B / (A + B)

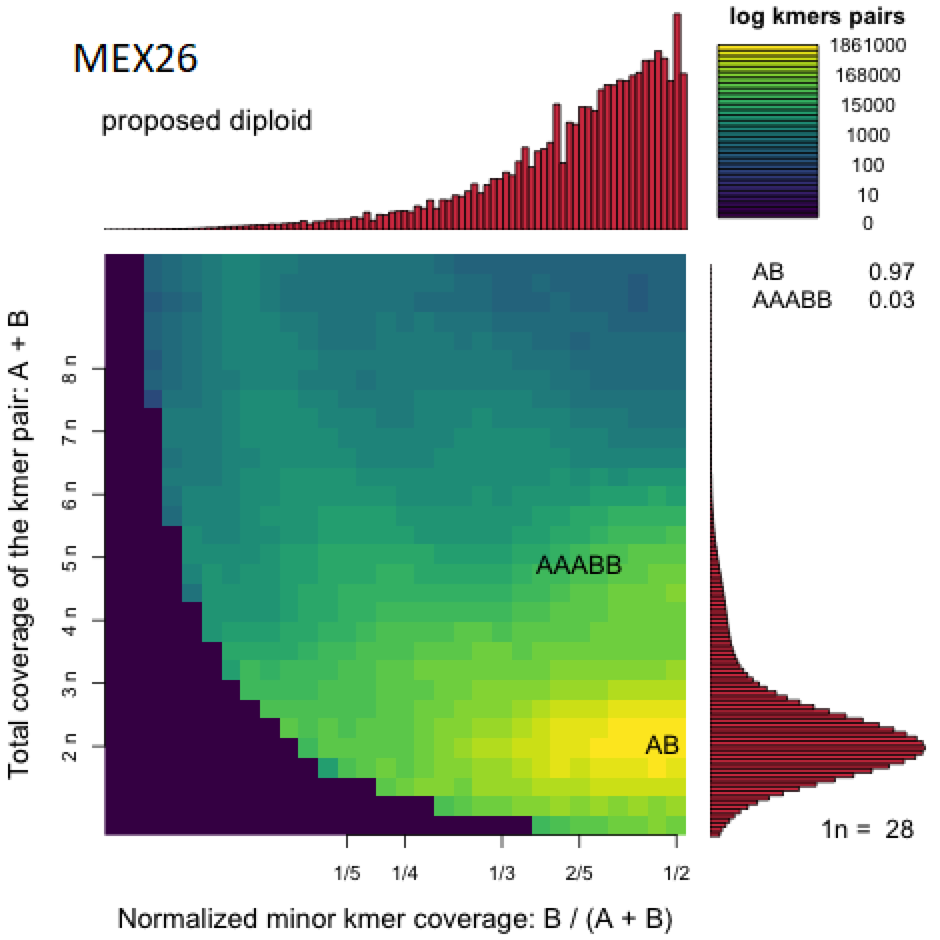
MEX26
proposed diploid
log kmers pairs
1861000
168000
15000
1000
100
10
0
AB 0.97
AAABB 0.03
AAABB
AB
1n = 28
Total coverage of the kmer pair: A + B
2n
3n
4n
5n
6n
7n
8n
1/5
1/4
1/3
2/5
1/2
Normalized minor kmer coverage: B / (A + B)

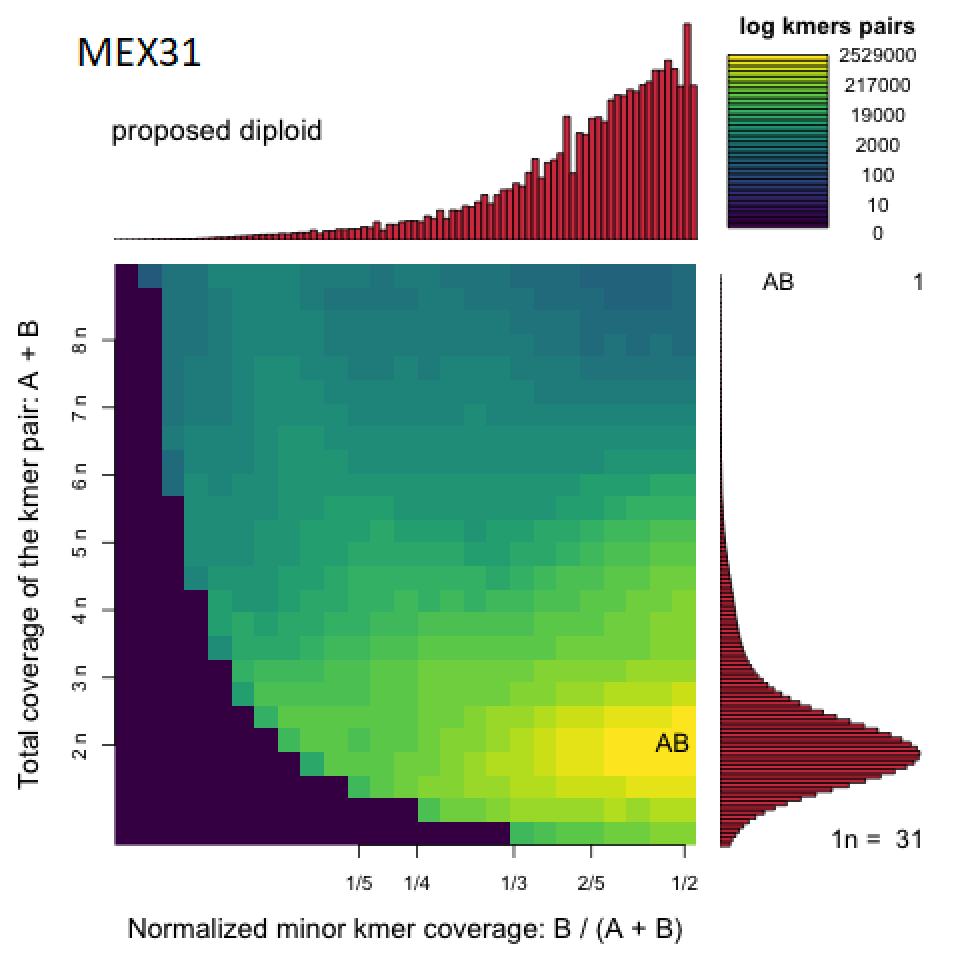
MEX31
proposed diploid
log kmers pairs
2529000
217000
19000
2000
100
10
0
AB
1
AB
Total coverage of the kmer pair: A + B
2n
3n
4n
5n
6n
7n
8n
1/5
1/4
1/3
2/5
1/2
1n = 31
Normalized minor kmer coverage: B / (A + B)

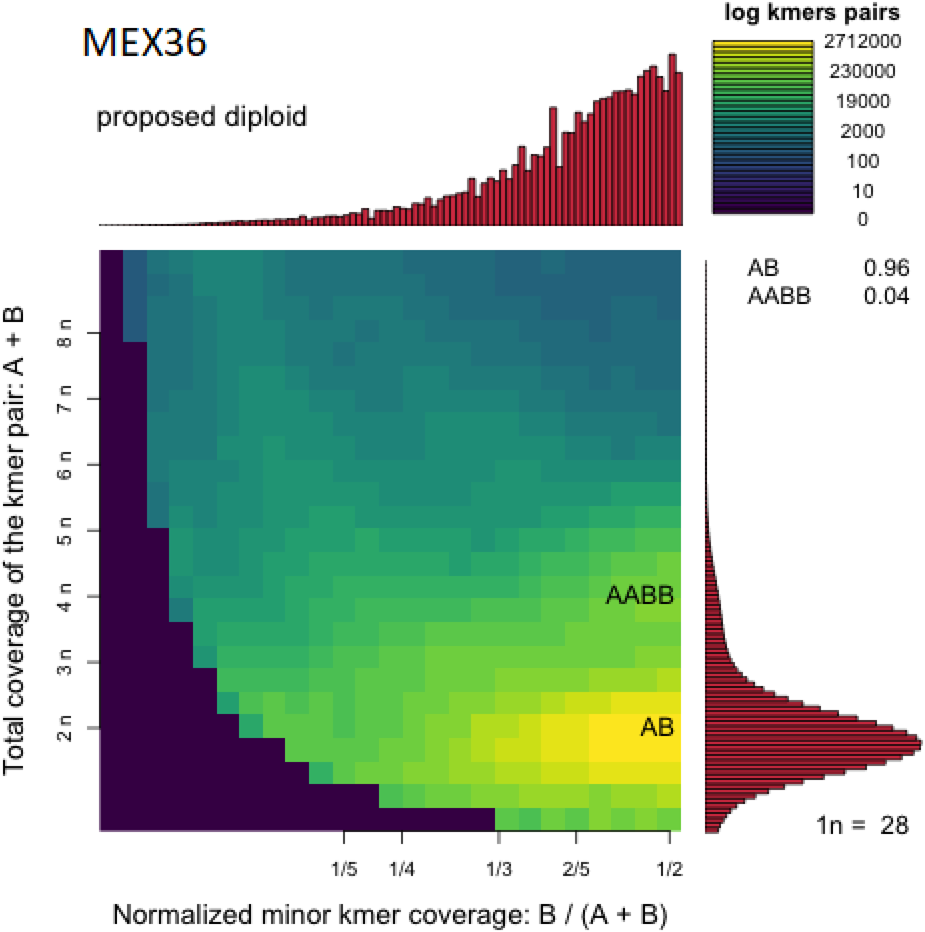

MEX36
proposed diploid
log kmers pairs
2712000
230000
19000
2000
100
10
0
AB 0.96
AABB 0.04
AABB
AB
1n = 28
Total coverage of the kmer pair: A + B
8n
7n
6n
5n
4n
3n
2n
1/5
1/4
1/3
2/5
1/2
Normalized minor kmer coverage: B / (A + B)

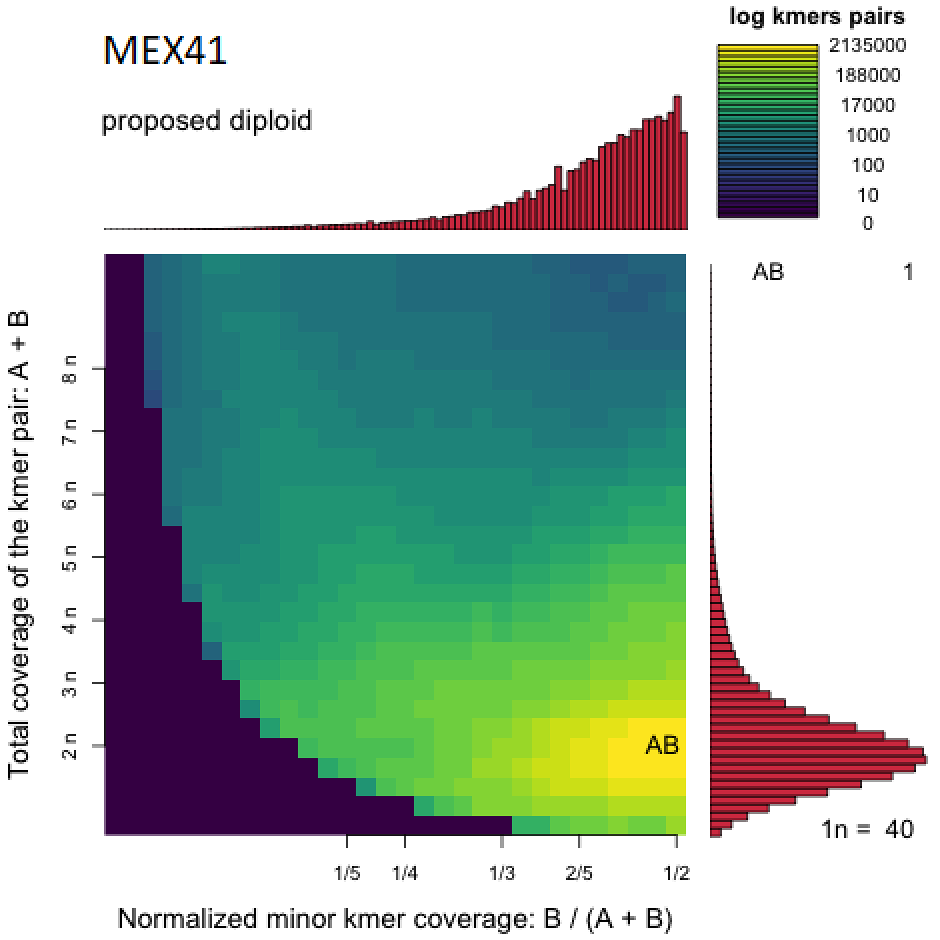
MEX41
proposed diploid
log kmers pairs
2135000
188000
17000
1000
100
10
0
AB
1
AB
1n = 40
Total coverage of the kmer pair: A + B
2n
3n
4n
5n
6n
7n
8n
1/5
1/4
1/3
2/5
1/2
Normalized minor kmer coverage: B / (A + B)

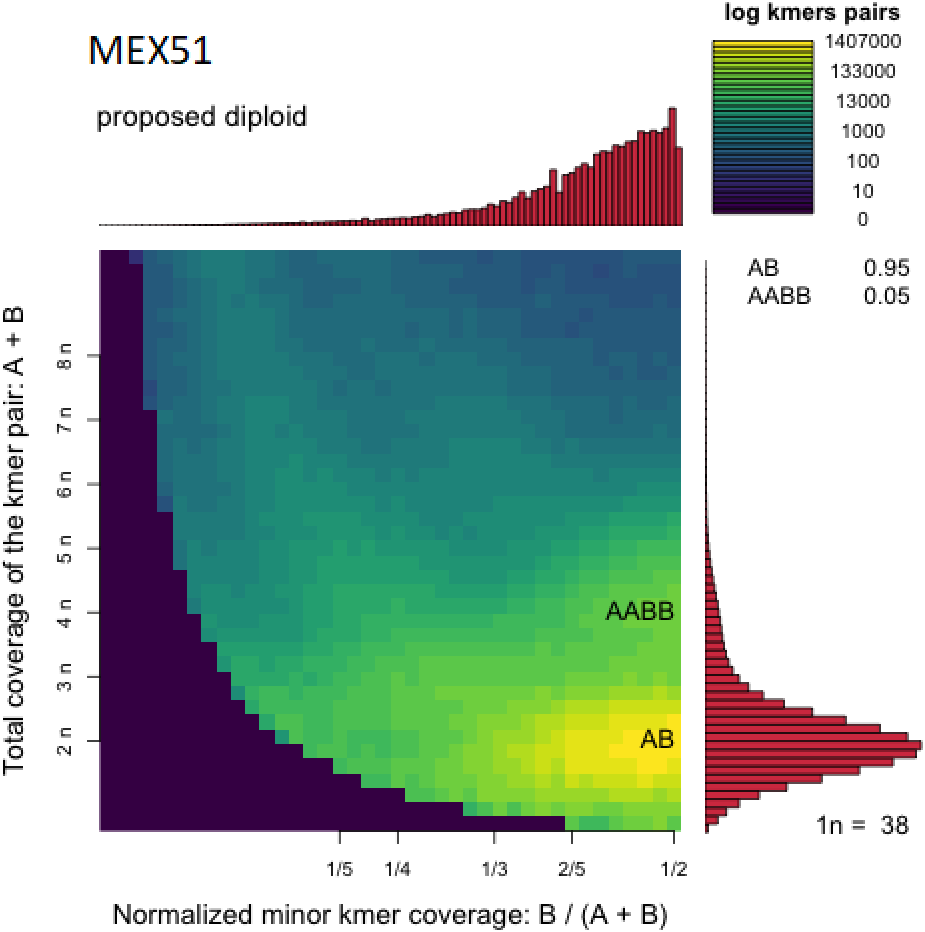

MEX51
proposed diploid
log kmers pairs
1407000
133000
13000
1000
100
10
0
AB 0.95
AABB 0.05
AABB
AB
1n = 38
Total coverage of the kmer pair: A + B
2n
3n
4n
5n
6n
7n
8n
1/5
1/4
1/3
2/5
1/2
Normalized minor kmer coverage: B / (A + B)

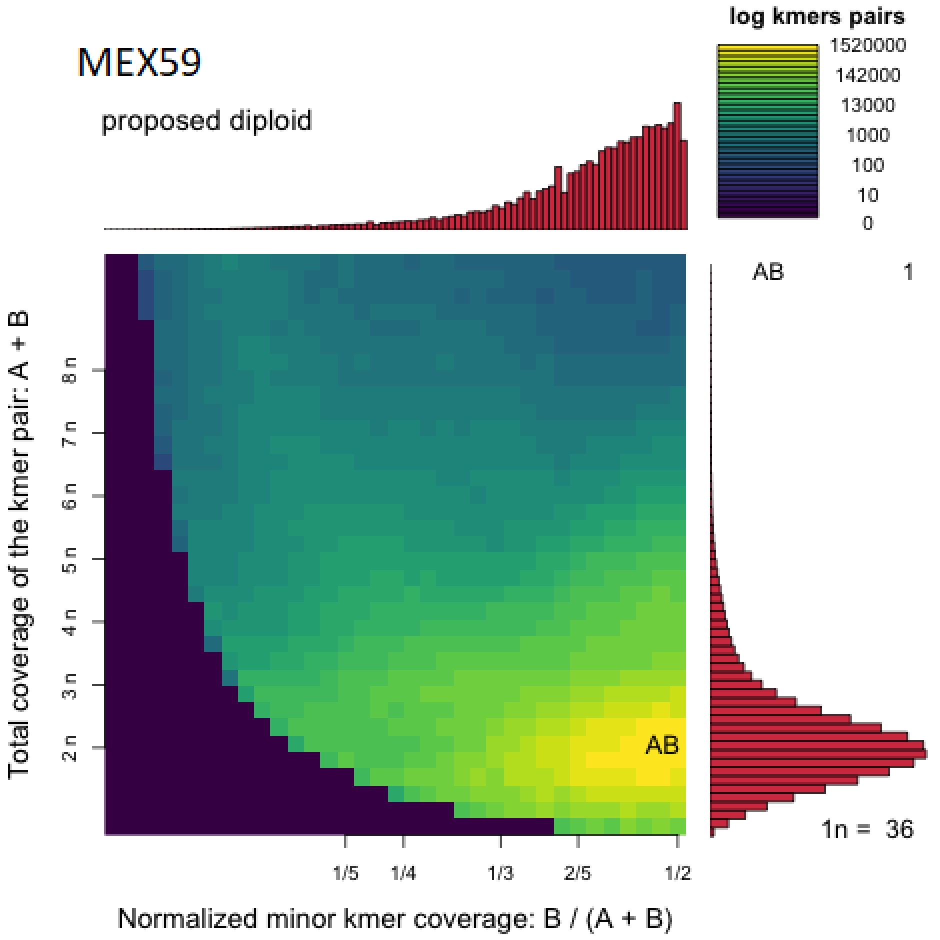

MEX59
proposed diploid
log kmers pairs
1520000
142000
13000
1000
100
10
0
AB
1
AB
1n = 36
Total coverage of the kmer pair: A + B
2n
3n
4n
5n
6n
7n
8n
1/5
1/4
1/3
2/5
1/2
Normalized minor kmer coverage: B / (A + B)

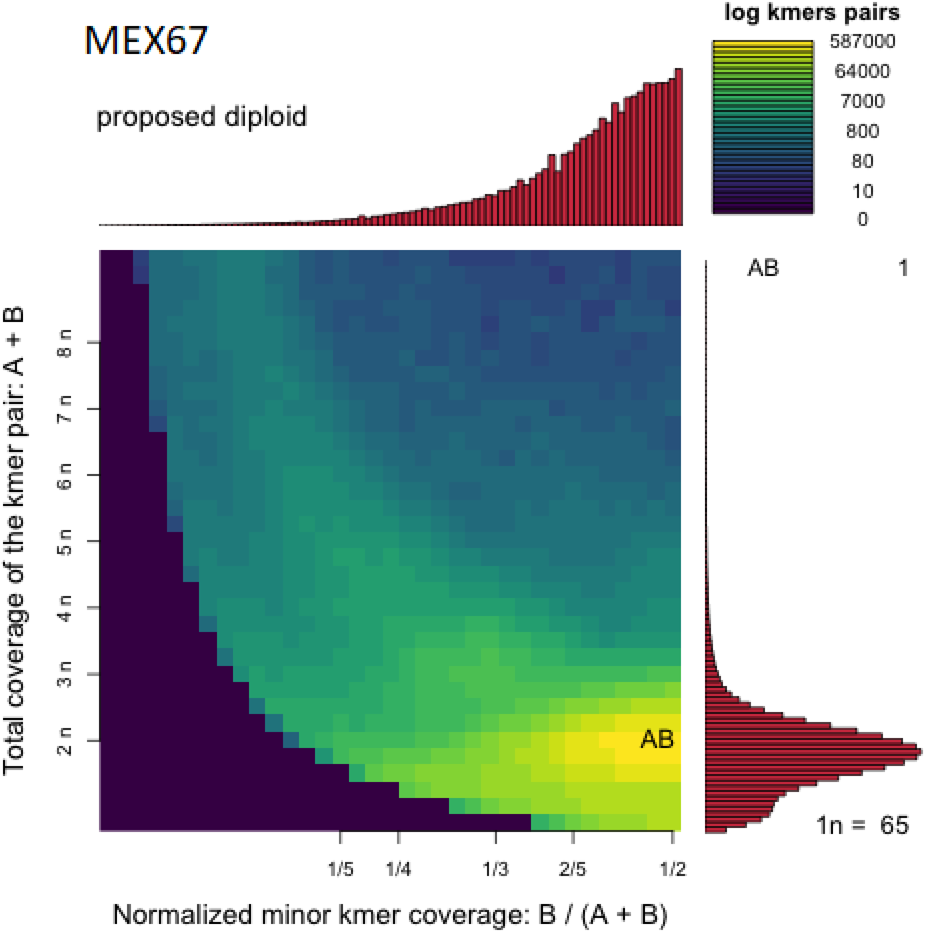
MEX67
proposed diploid
log kmers pairs
587000
64000
7000
800
80
10
0
AB
1
AB
1n = 65
Total coverage of the kmer pair: A + B
2n
3n
4n
5n
6n
7n
8n
1/5
1/4
1/3
2/5
1/2
Normalized minor kmer coverage: B / (A + B)

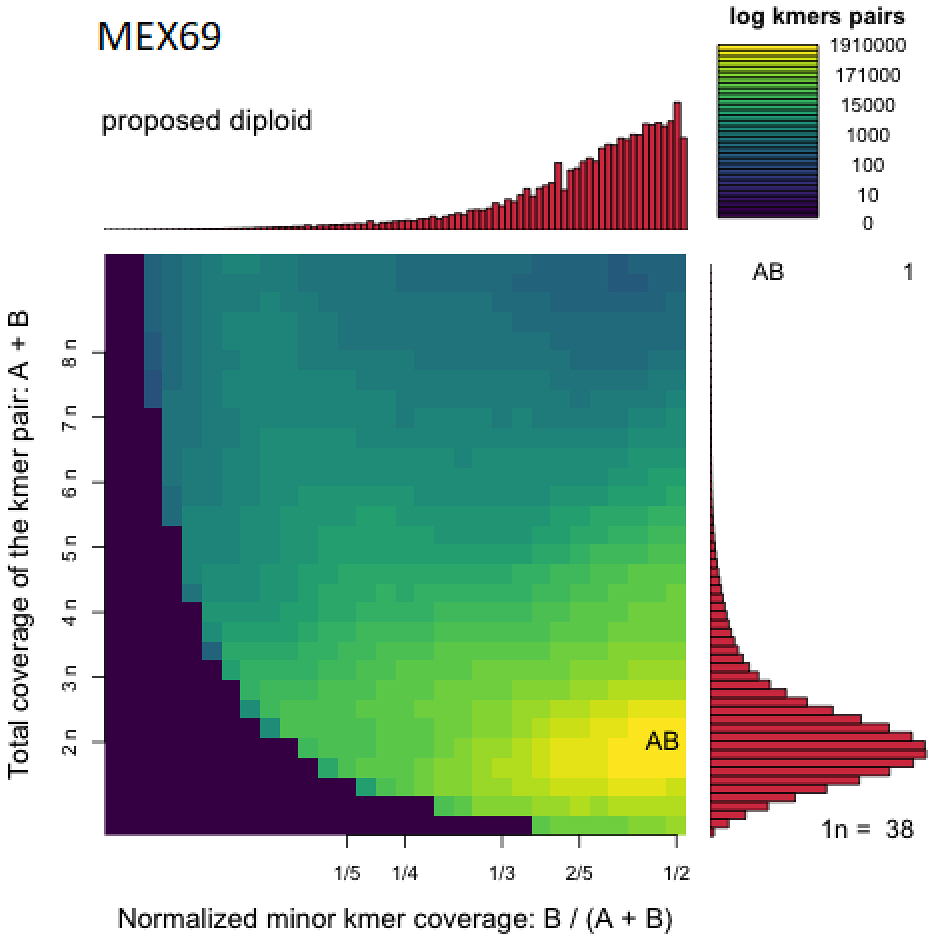
MEX69
proposed diploid
log kmers pairs
1910000
171000
15000
1000
100
10
0
AB
1
AB
1n = 38
Total coverage of the kmer pair: A + B
2n
3n
4n
5n
6n
7n
8n
1/5
1/4
1/3
2/5
1/2
Normalized minor kmer coverage: B / (A + B)

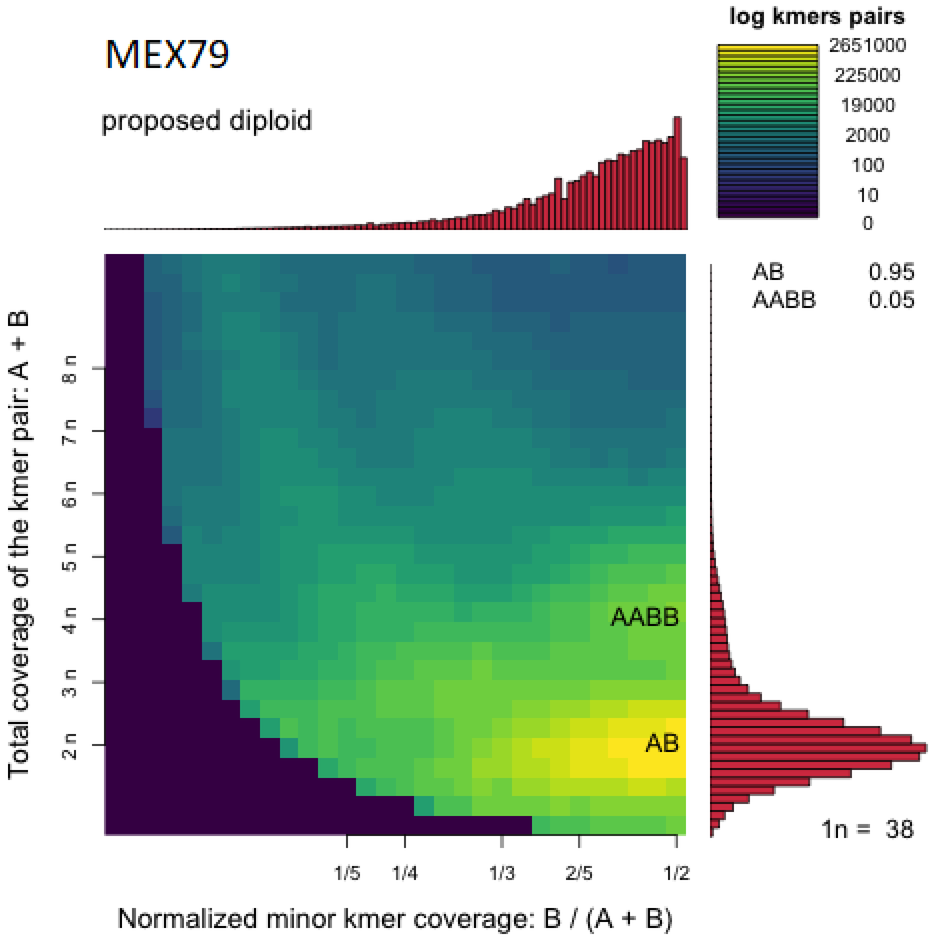
MEX79
proposed diploid
log kmers pairs
2651000
225000
19000
2000
100
10
0
AB 0.95
AABB 0.05
AABB
AB
1n = 38
Total coverage of the kmer pair: A + B
2n
3n
4n
5n
6n
7n
8n
1/5
1/4
1/3
2/5
1/2
Normalized minor kmer coverage: B / (A + B)
