# Supplementary figures and images for: Genomic Insights into Cultivated Mexican Vanilla planifolia Reveal High Levels of Heterozygosity Stemming from Hybridization
Source: Plants (Basel). 2022 Aug 11;11(16):2090. doi: 10.3390/plants11162090 (PMC9412680; doi:10.3390/plants11162090)

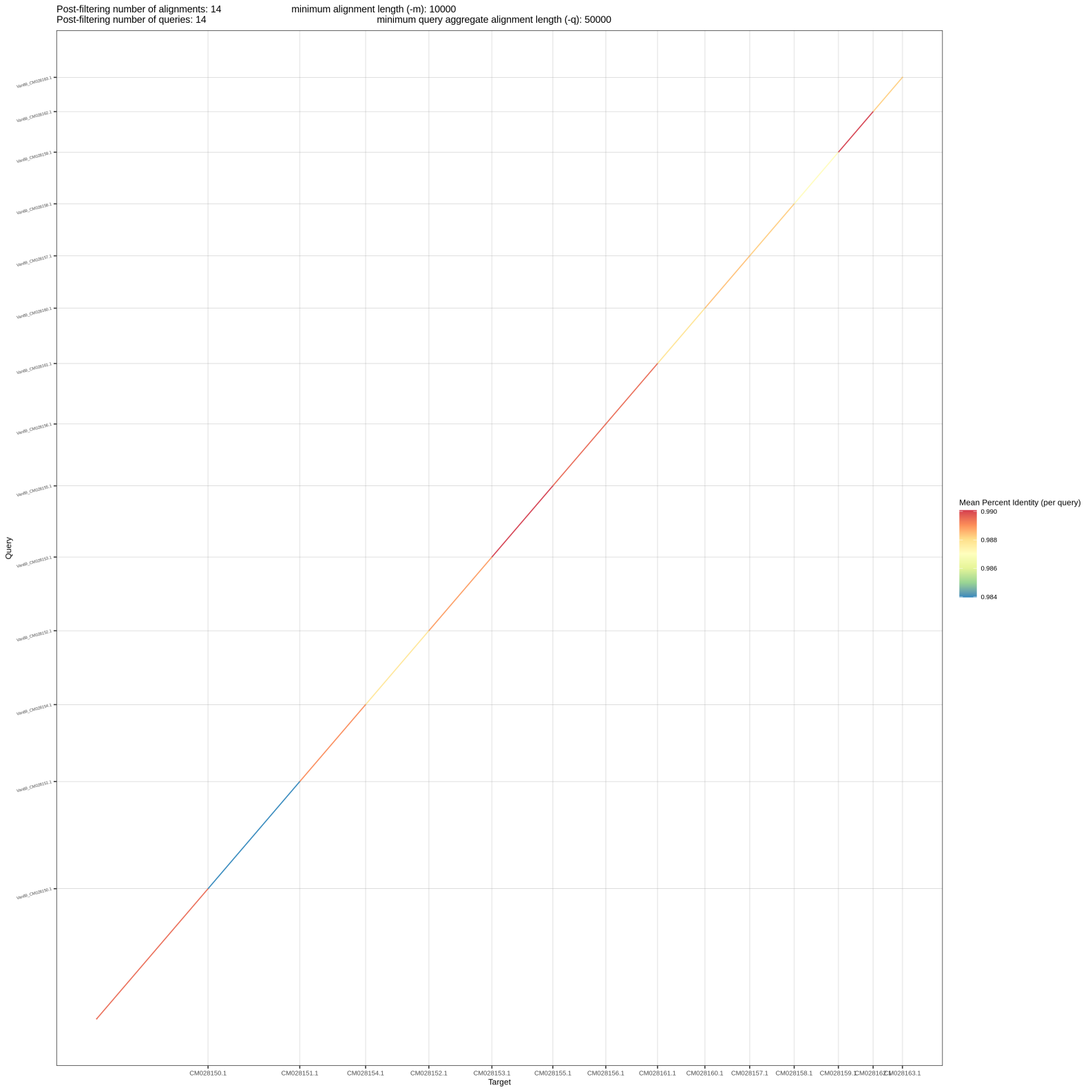

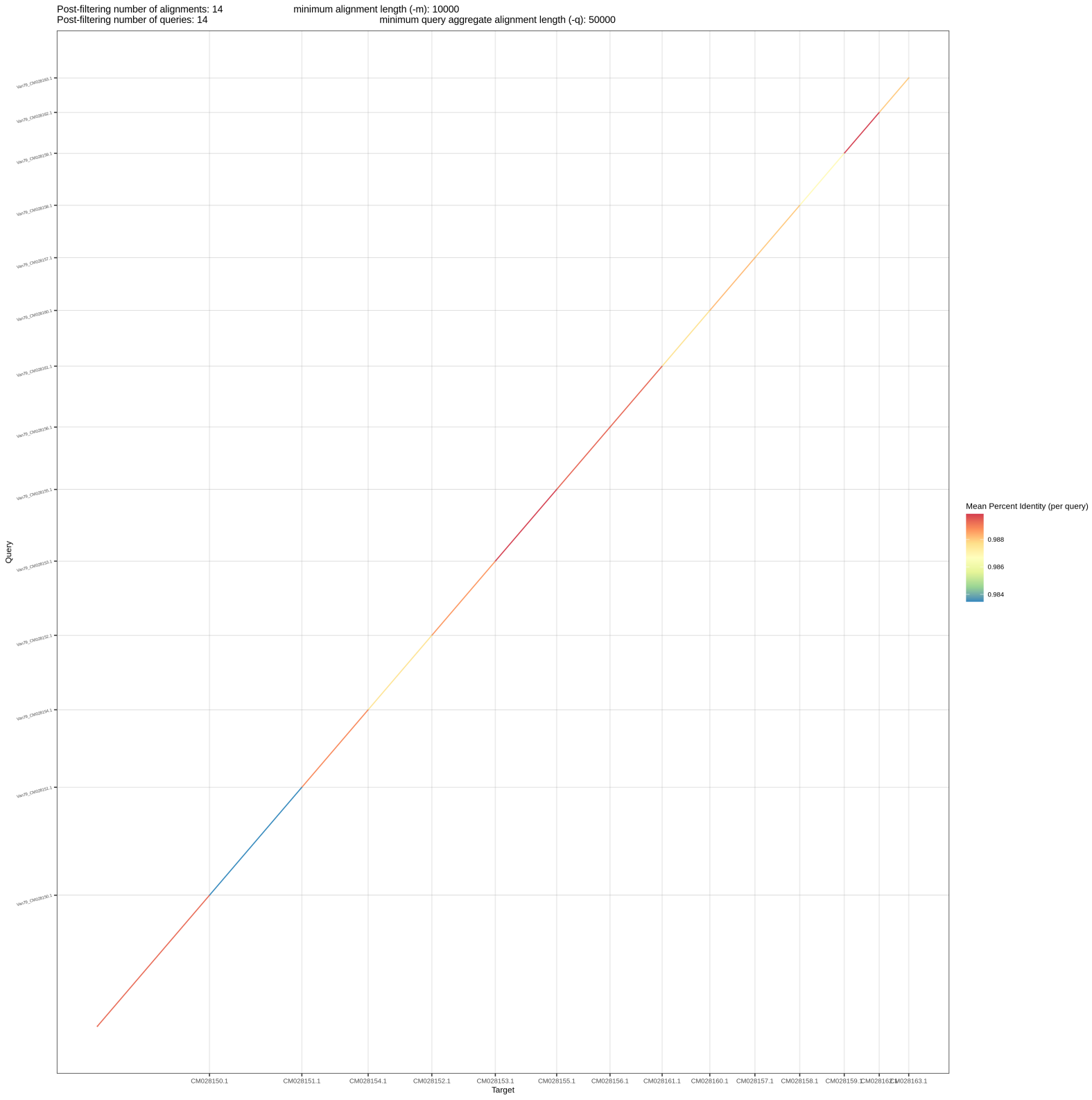

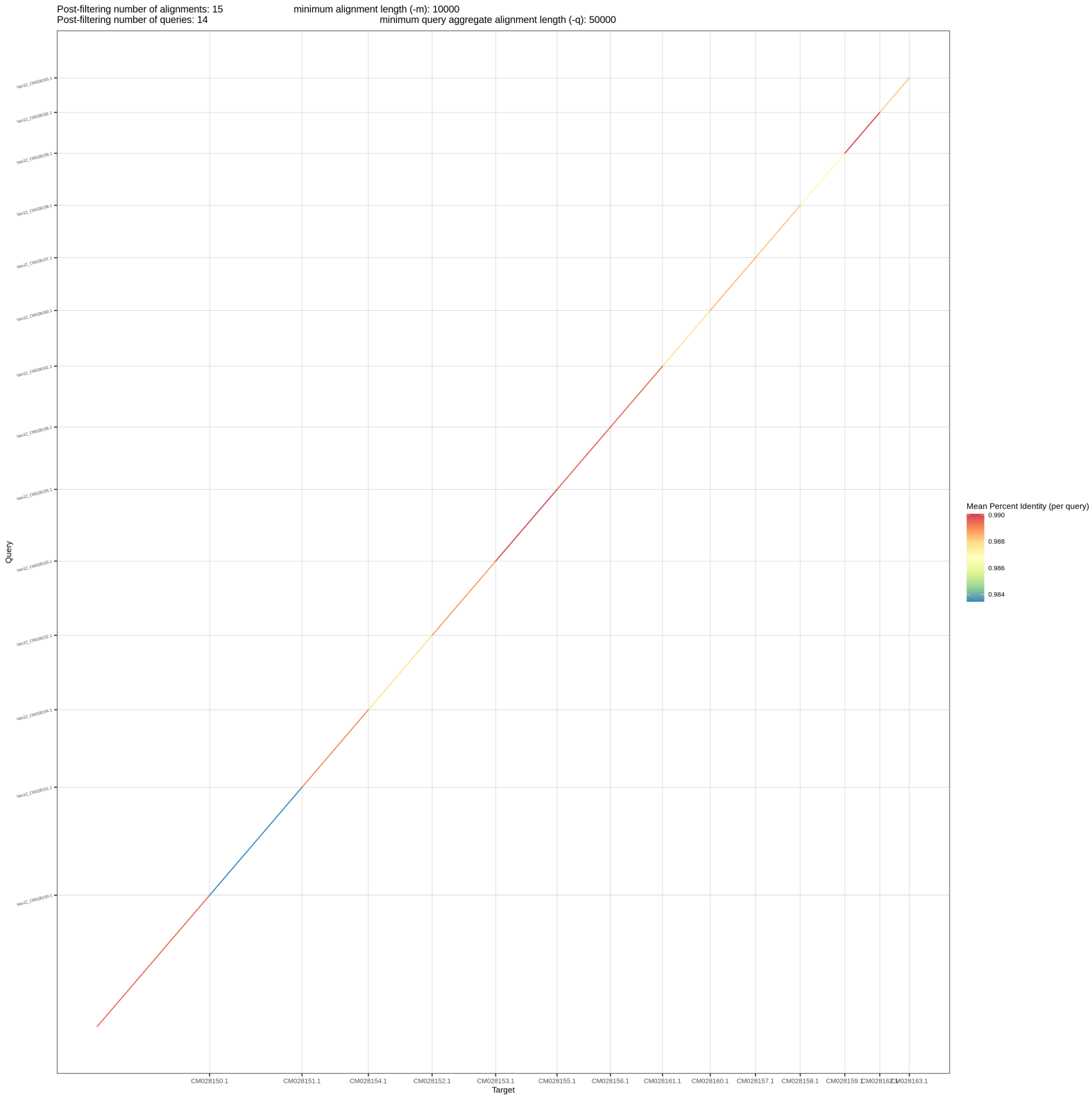

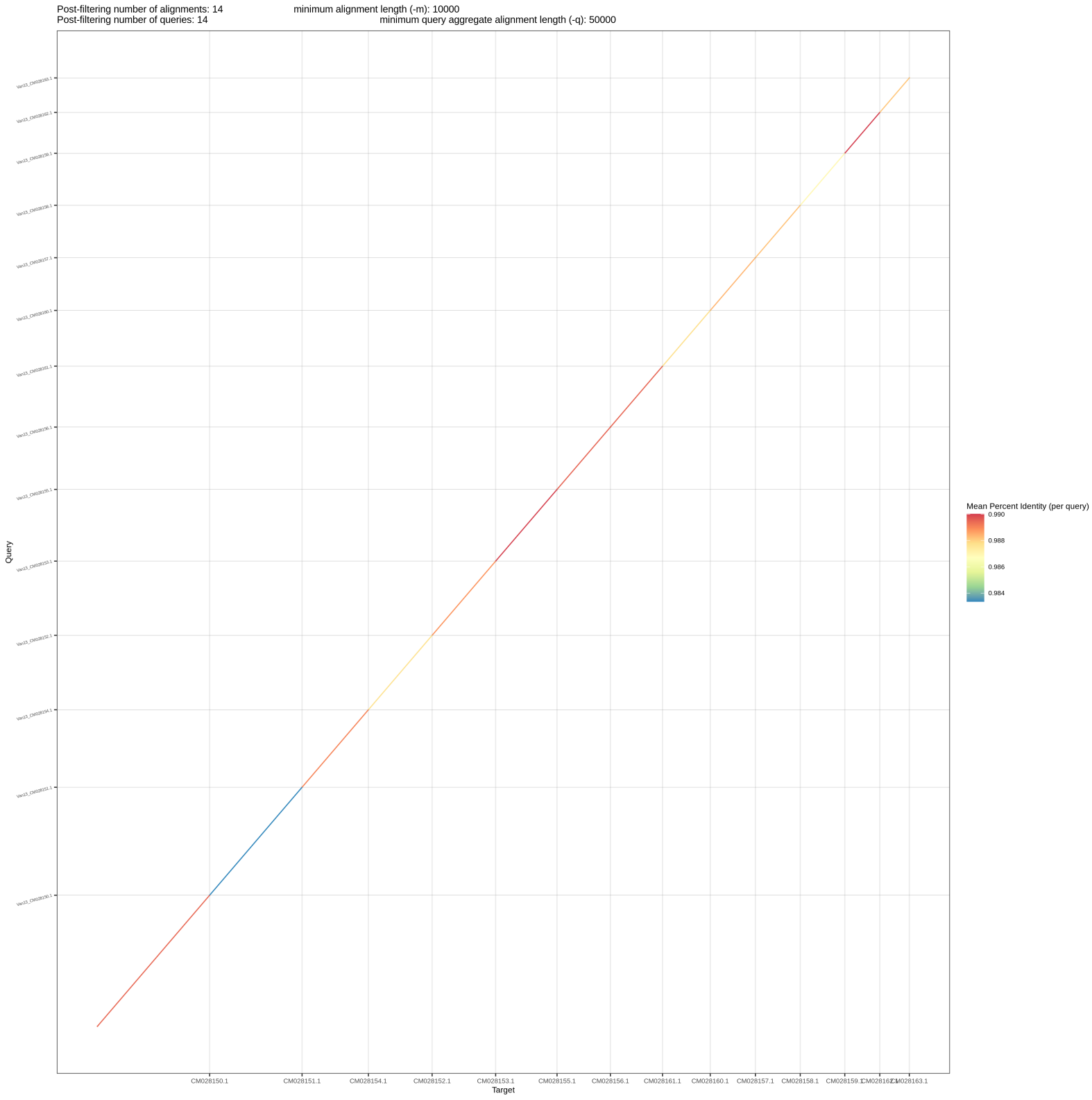

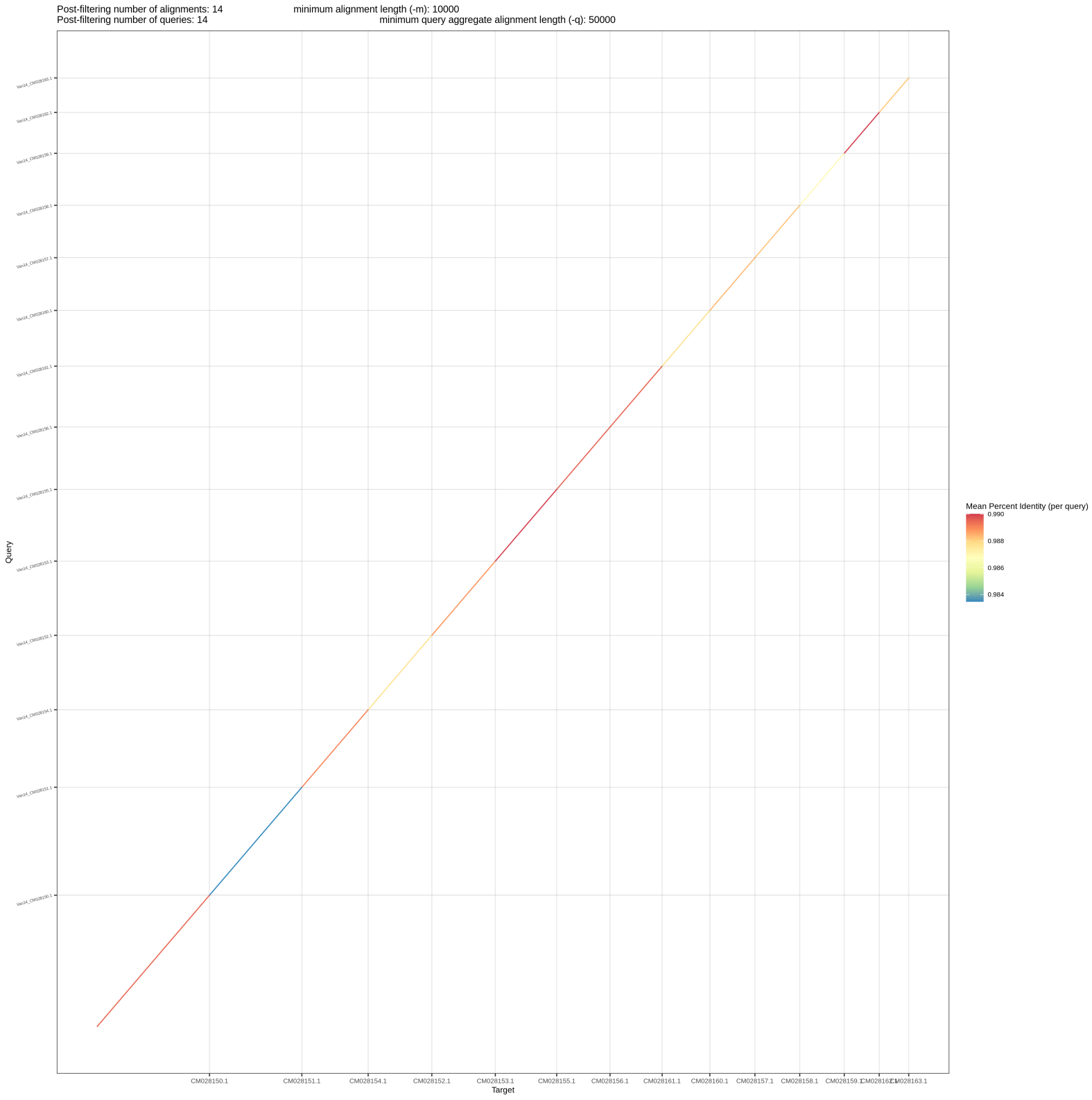

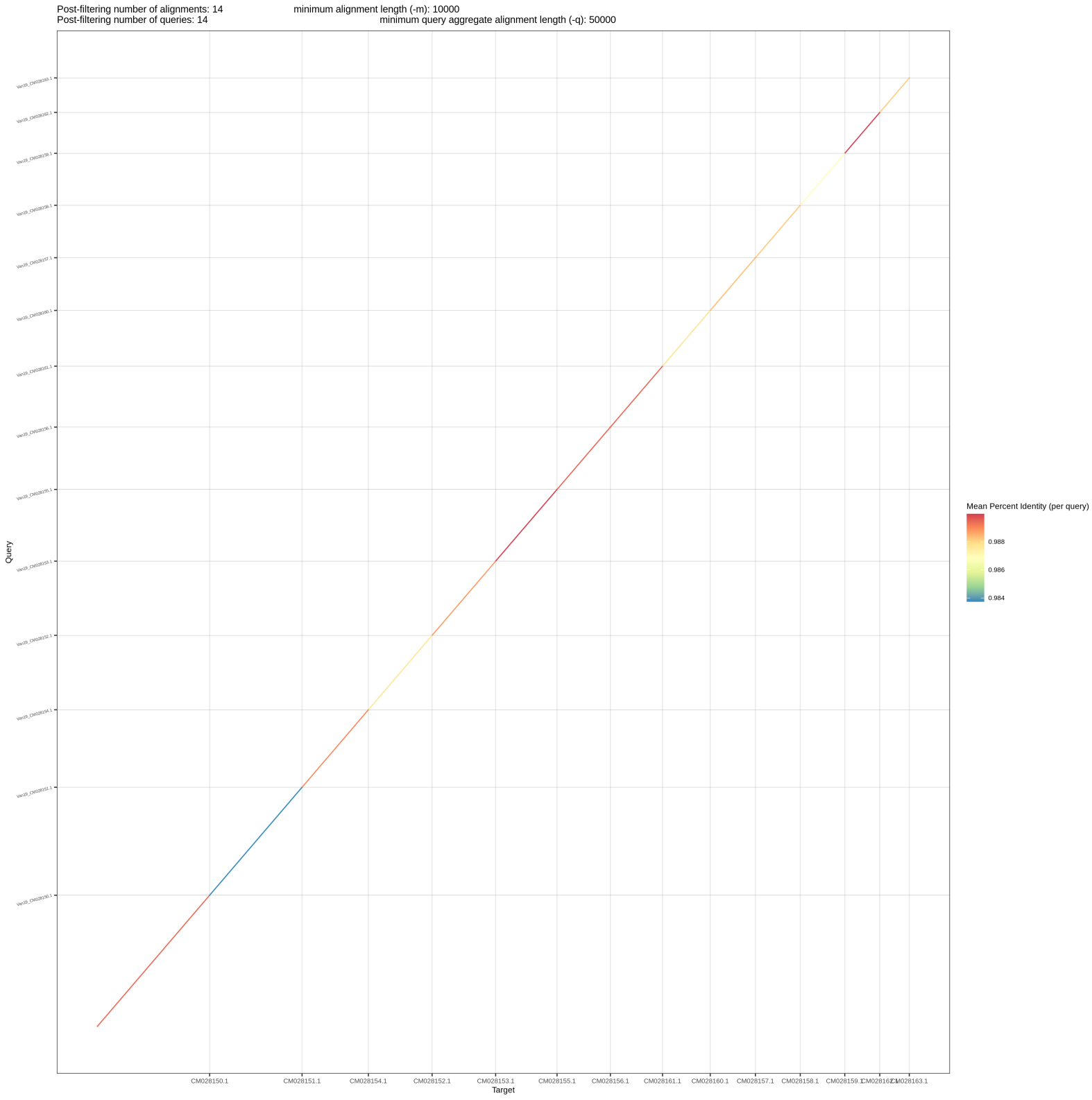

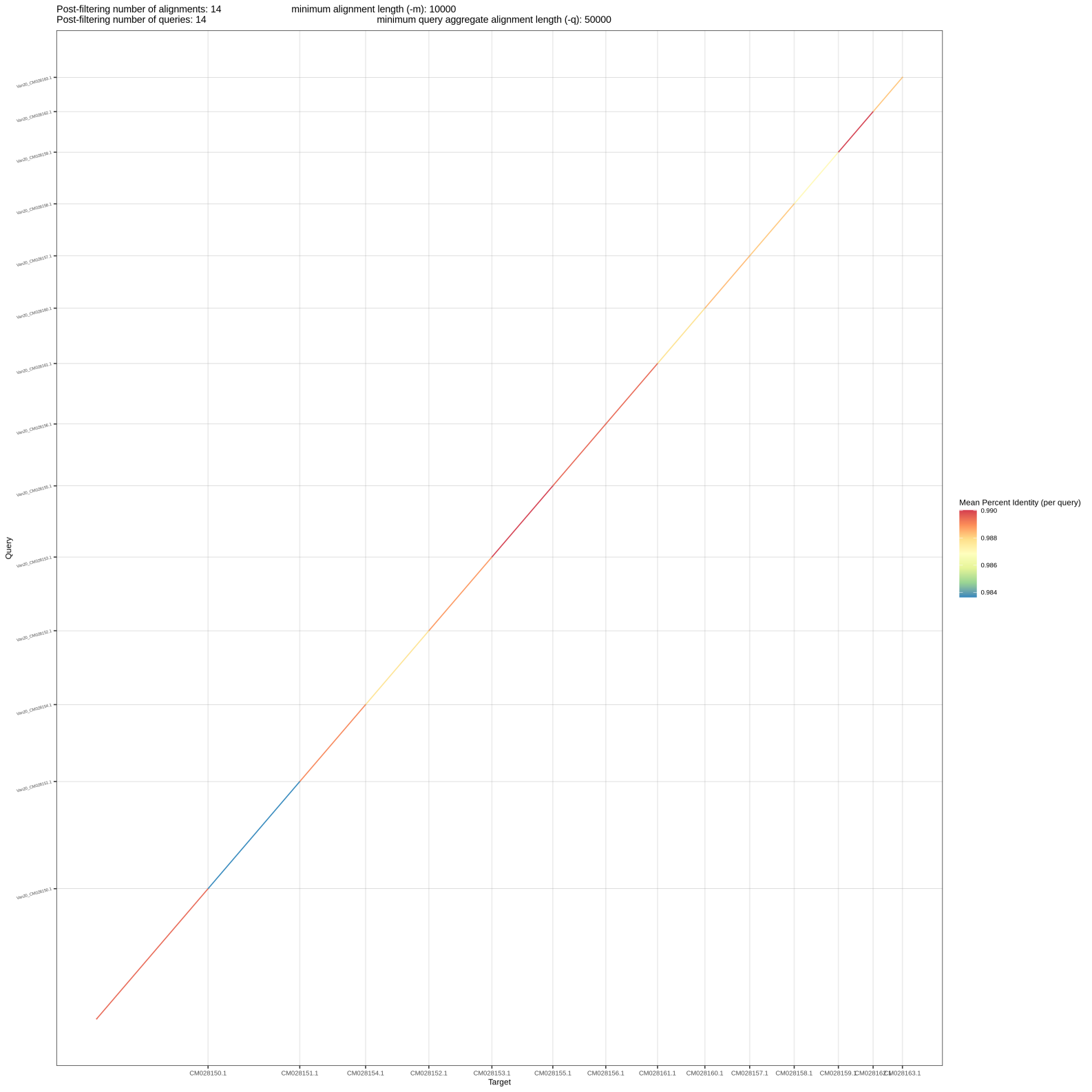

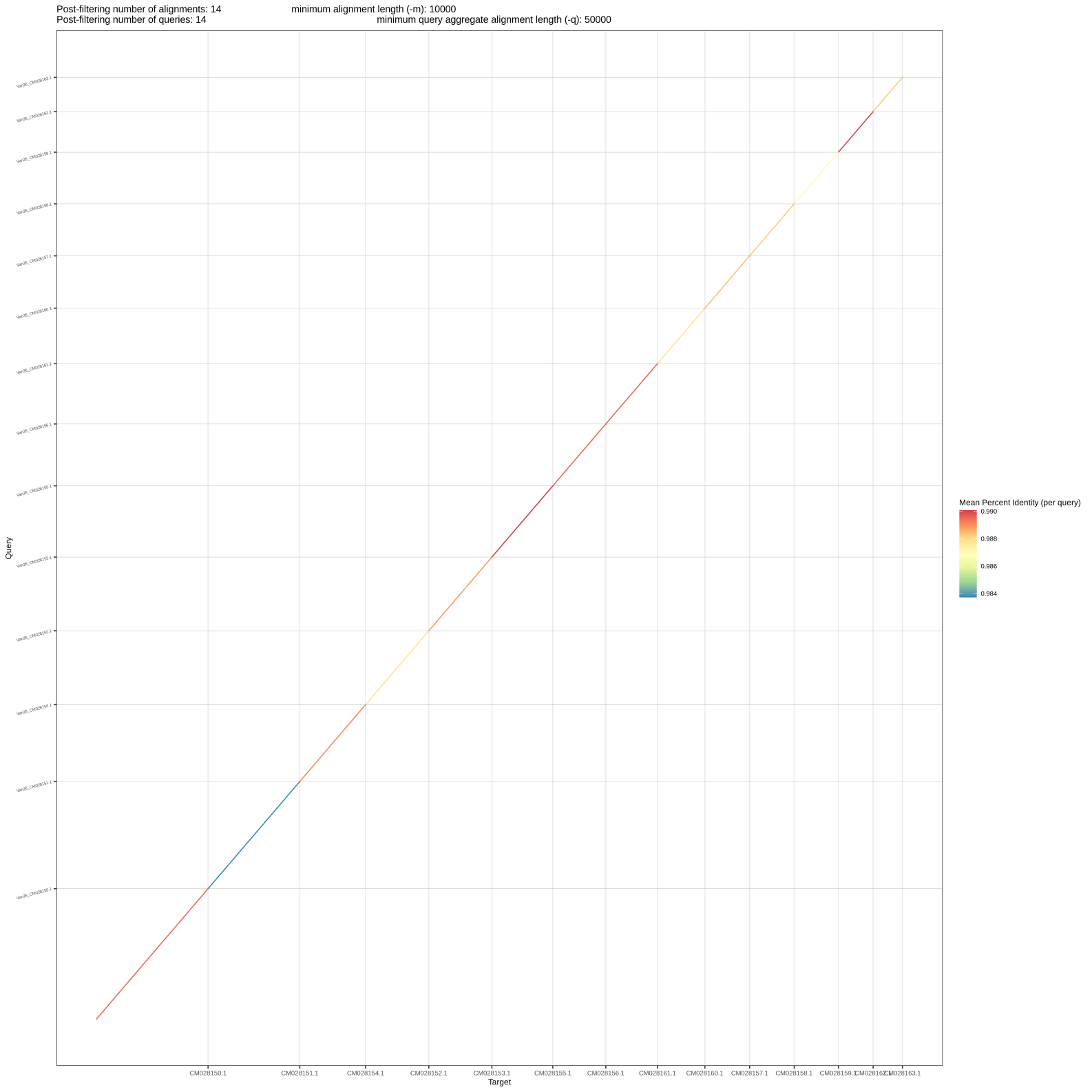

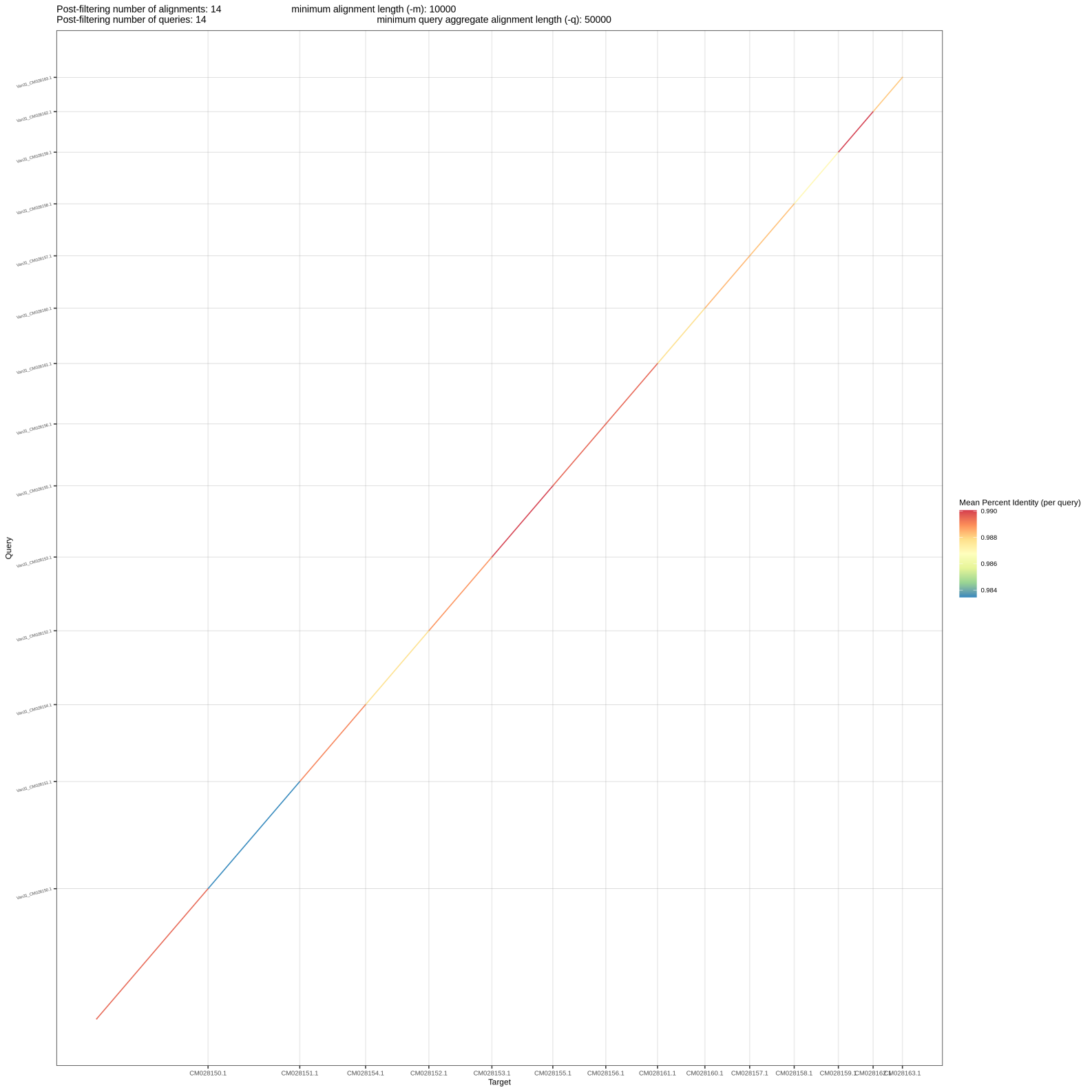

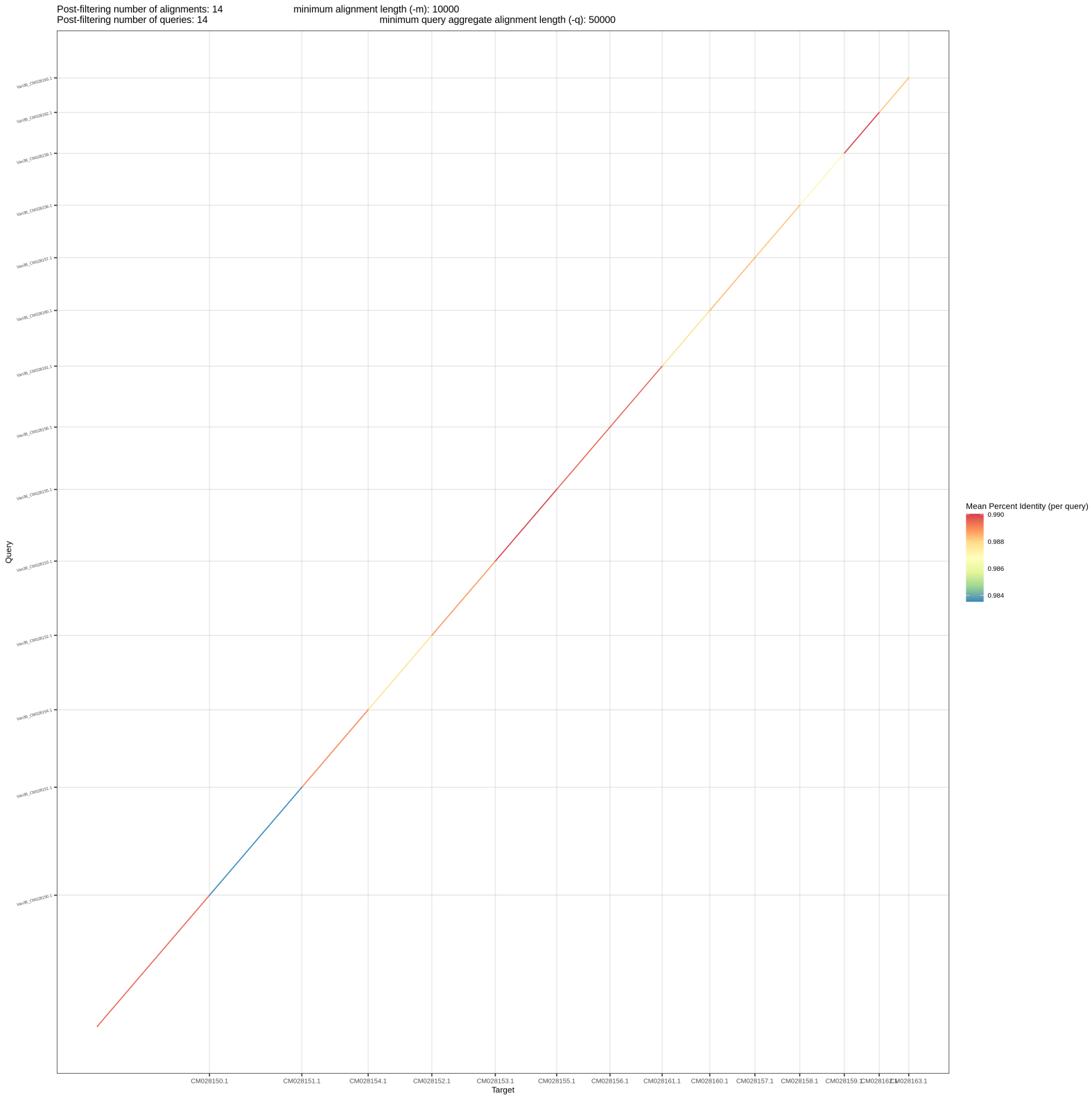

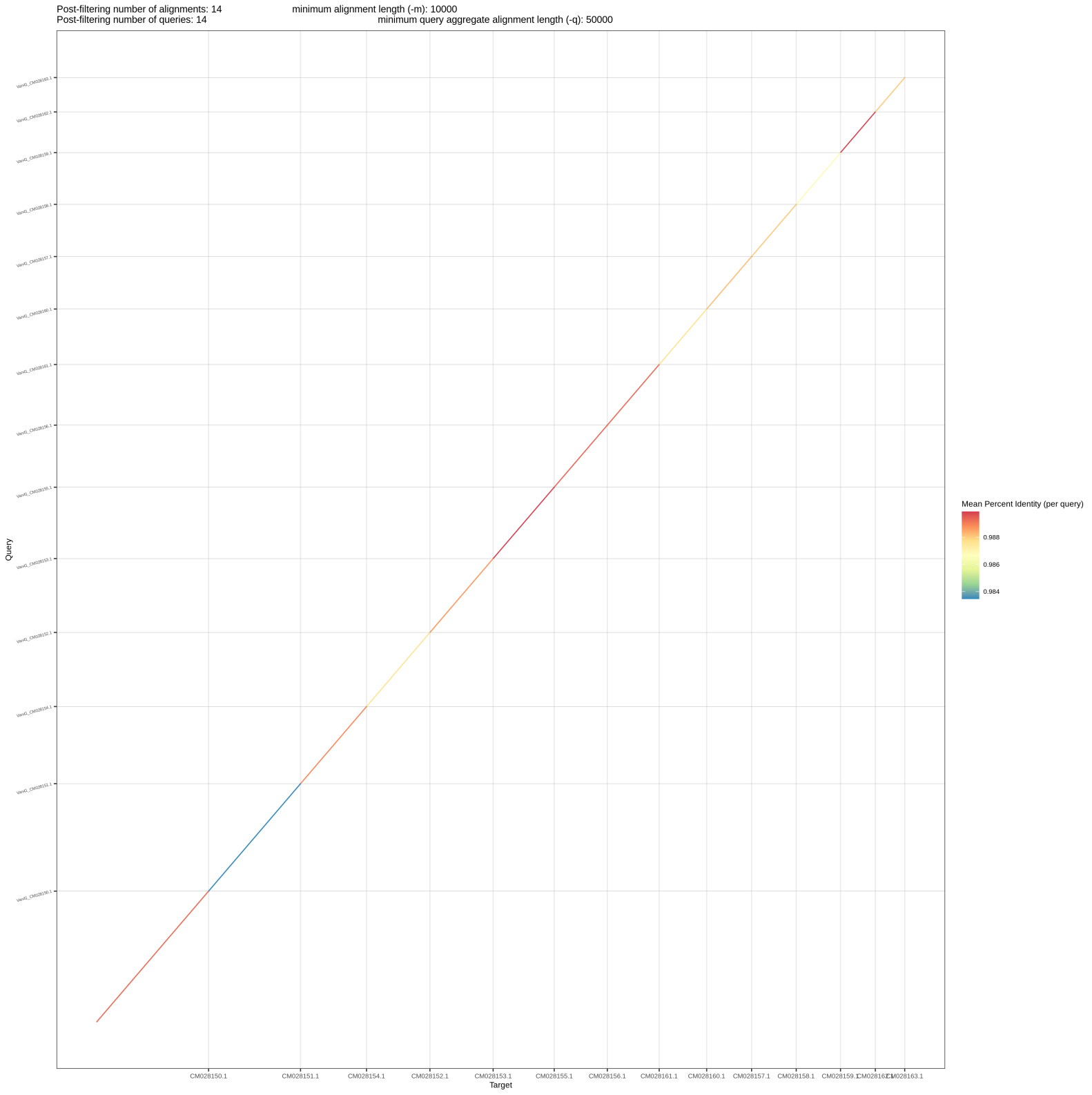

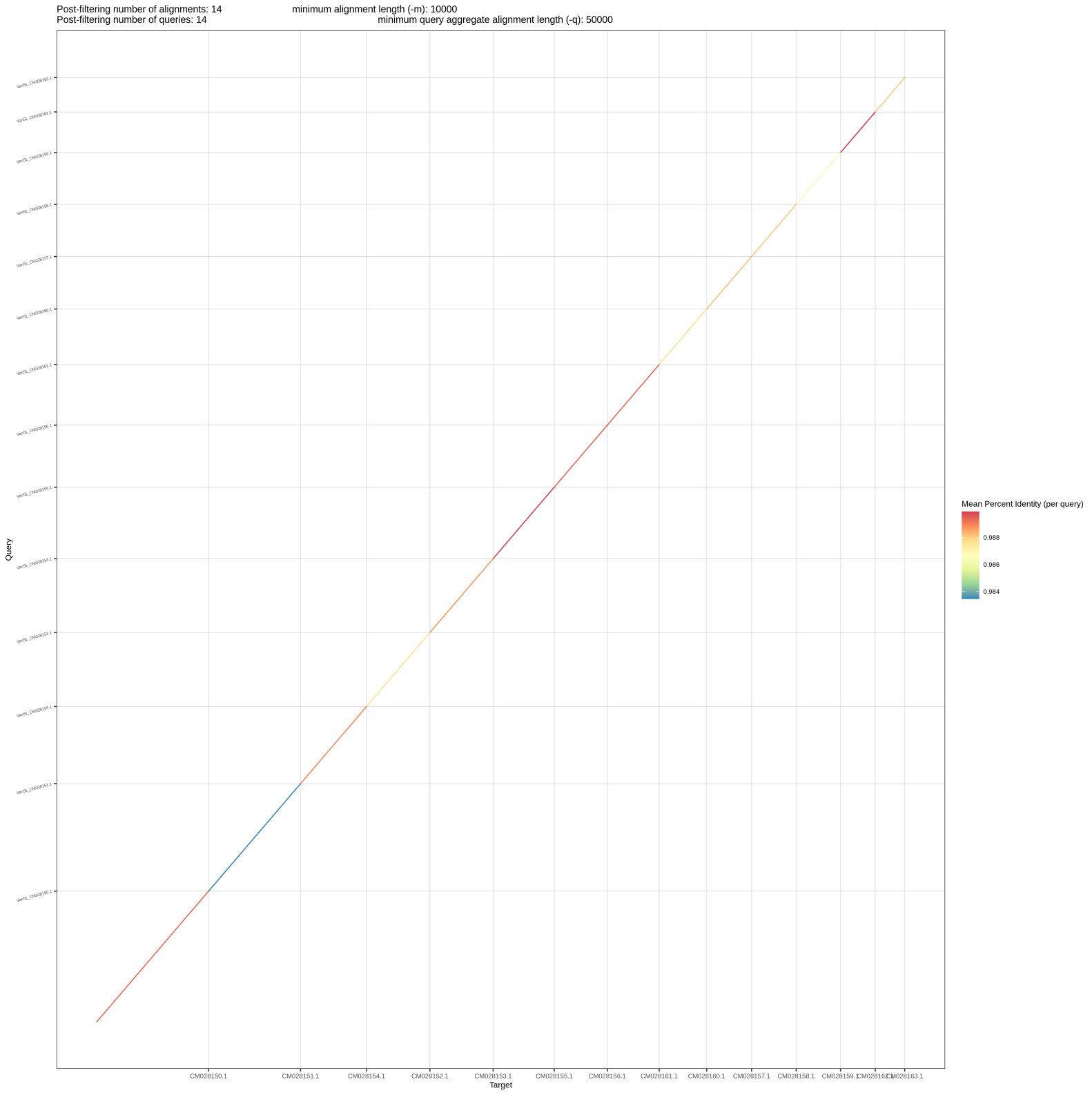

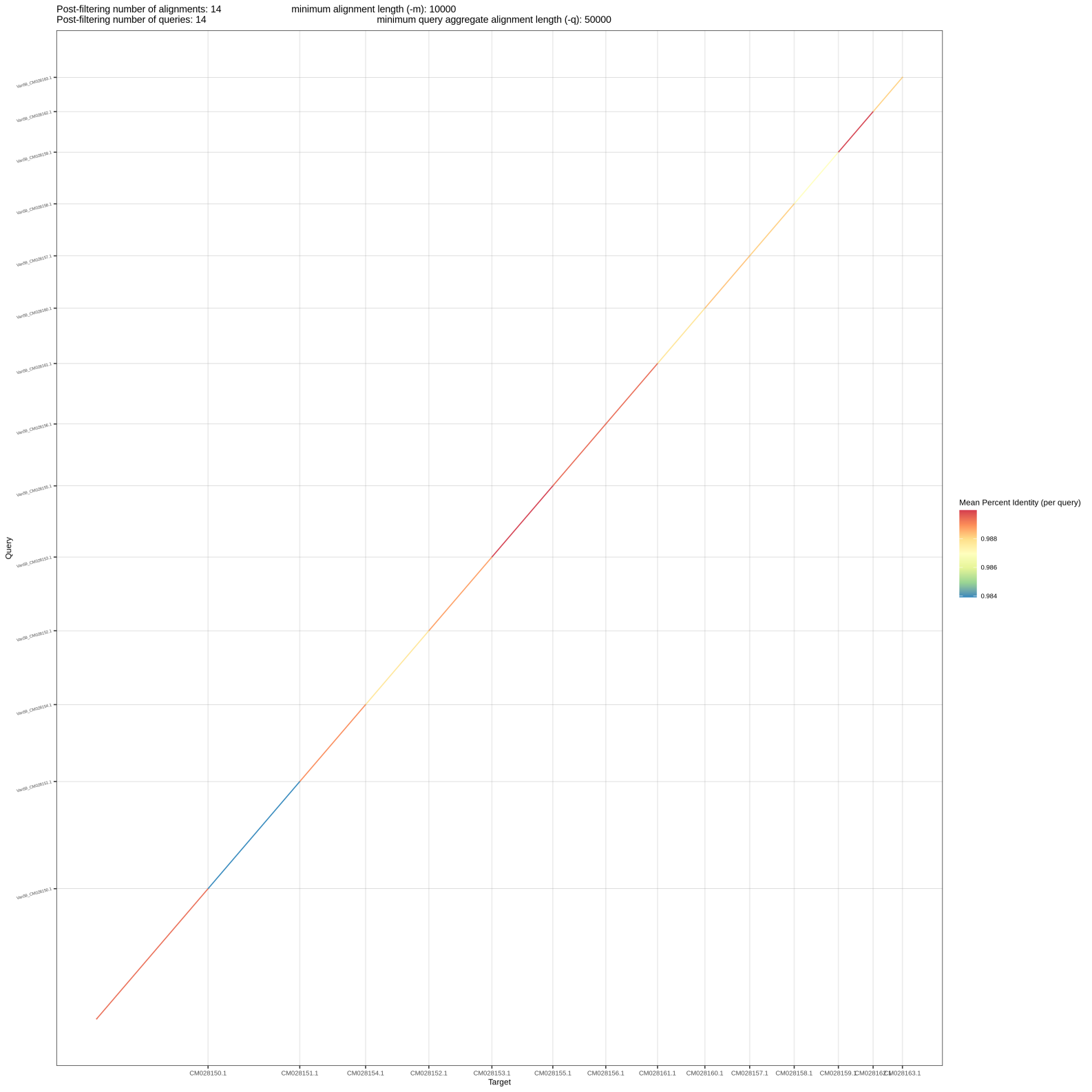

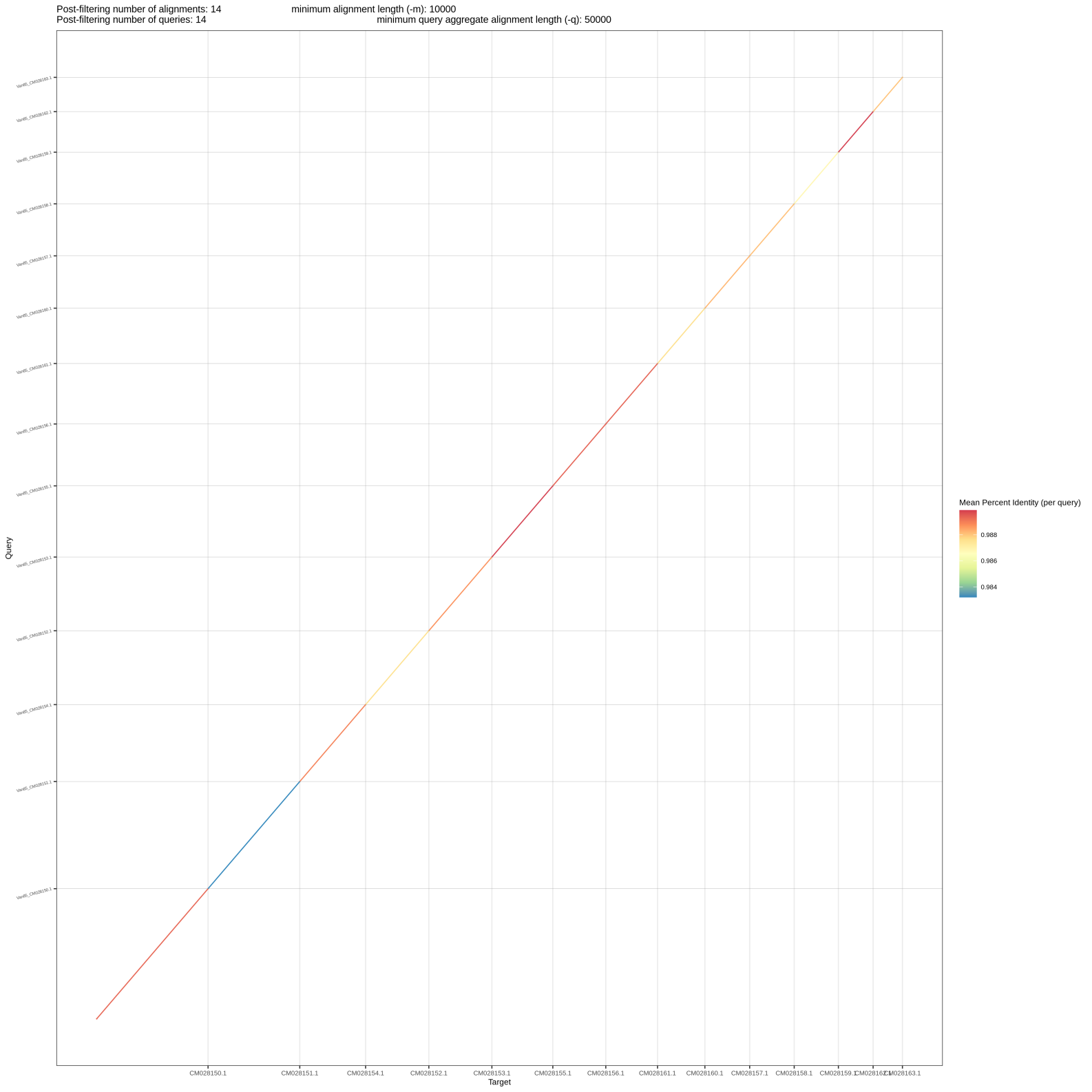

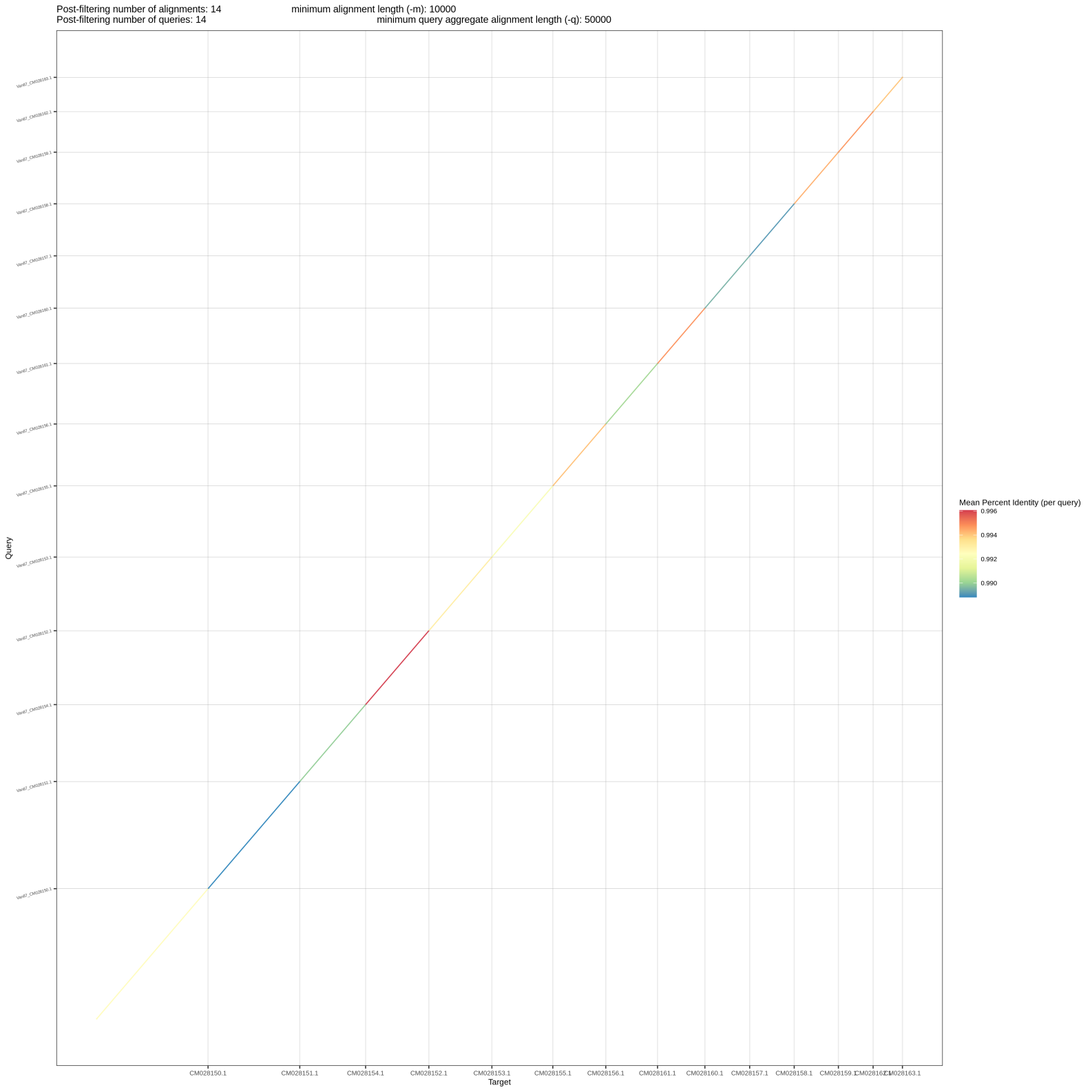

Supplement: Supplementary file 1 [file plants-11-02090-s001.zip › Figure S3.pdf]

A.

LD threshold of 0.8 resulting in 419885 SNPs

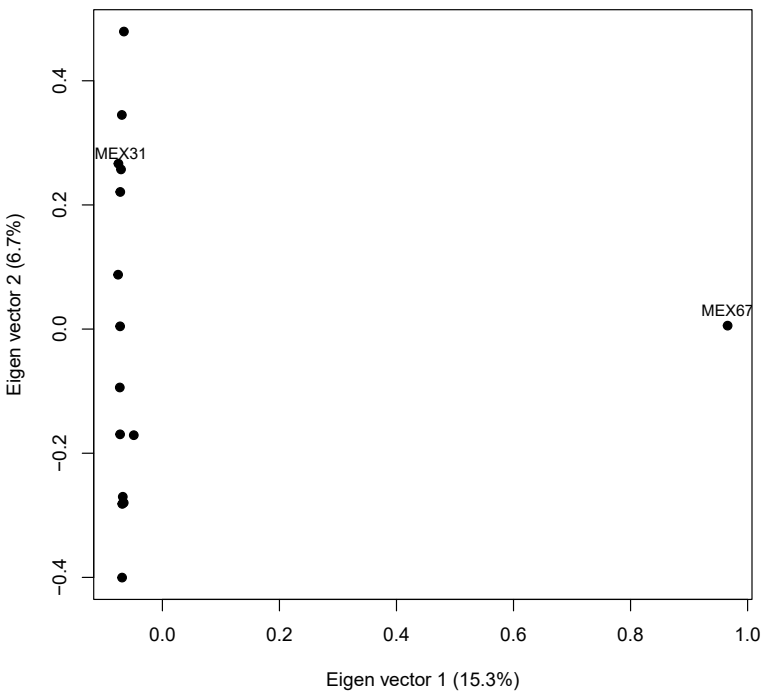

B.

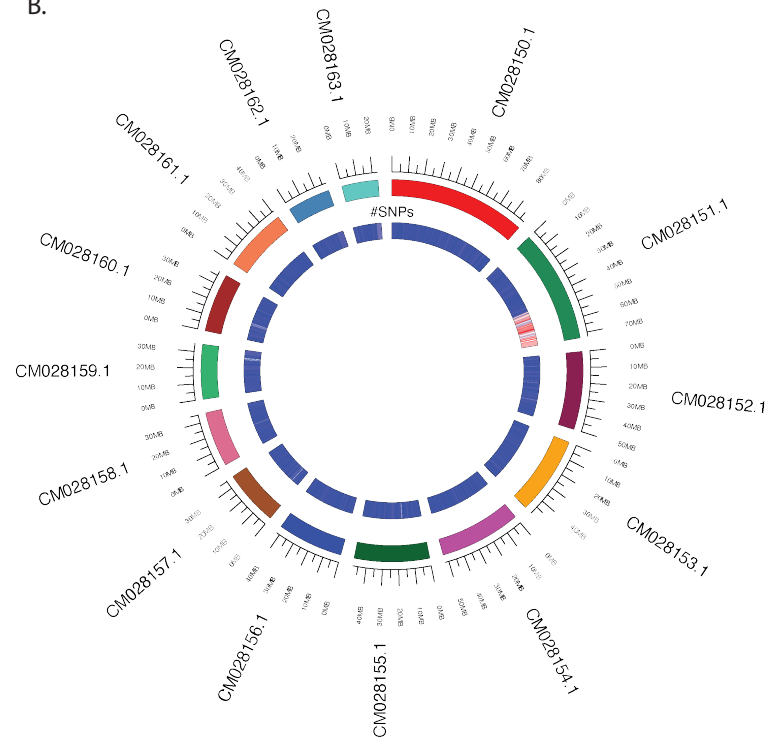

Supplement: Supplementary file 1 [file plants-11-02090-s001.zip › Figure S4.pdf]
